# Targeting HMGCS2: Ketogenesis Suppression Accelerates NAFLD Progression in T2DM Comorbidity, While Cynaroside Ameliorates NASH in Concomitant T2DM

**DOI:** 10.3390/biom15081181

**Published:** 2025-08-18

**Authors:** Yongsheng Shu, Wanqing Shen, Wanyu Feng, Meijun Pan, Xinyi Xu, Shuguo Zheng, Huanhuan Jin

**Affiliations:** 1 Department of Pharmacology, School of Pharmacy, Wannan Medical College, Wuhu 241002, China; 20249138@stu.wnmc.edu.cn (Y.S.); 20229074@stu.wnmc.edu.cn (W.S.); 20239135@stu.wnmc.edu.cn (W.F.); 20239130@stu.wnmc.edu.cn (M.P.); 22107080042@stu.wnmc.edu.cn (X.X.); 2 Laboratory of Pharmacology of Chinese Medicine, School of Pharmacy, Wannan Medical College, Wuhu 241002, China

**Keywords:** non-alcoholic fatty liver disease, type 2 diabetes mellitus, ketogenesis, cynaroside, cellular senescence, hydroxymethylglutaryl-CoA synthase 2

## Abstract

Patients with concurrent non-alcoholic fatty liver disease (NAFLD) and type 2 diabetes mellitus (T2DM) exhibit increased susceptibility to non-alcoholic steatohepatitis (NASH), advanced hepatic fibrosis, cirrhosis, and hepatocellular carcinoma. This study investigated the contribution of ketogenesis to T2DM-mediated NAFLD exacerbation and elucidated the therapeutic mechanism of cynaroside in NASH-complicated T2DM. Male C57BL/6J mice were given CDAHFD combined with streptozotocin to establish stage-specific NAFLD with T2DM models. Hepatic HMGCS2 expression was modulated via tail vein injection of adenoviral vectors for HMGCS2 overexpression or knockdown. Cynaroside was administered orally from week 5 to week 8. The results showed that concurrent T2DM accelerated NAFLD progression, accompanied by a dysregulated ketogenesis that was correlated with disease severity. Hepatic HMGCS2 expression paralleled circulating ketone body concentrations, indicating that HMGCS2-mediated ketogenic dysregulation contributed to NAFLD pathogenesis in T2DM contexts. HMGCS2 overexpression in NASH-T2DM models significantly attenuated steatohepatitis progression through the enhancement of ketogenesis. Cynaroside administration ameliorated hepatic pathology in NASH-T2DM mice by (1) reducing hepatocellular injury and lobular inflammation; (2) decreasing intrahepatic lipid accumulation; and (3) suppressing hepatocyte senescence and the secretion of SASP factors. Mechanistically, cynaroside exerted therapeutic effects via HMGCS2-mediated ketogenesis. Our data demonstrated that ketogenic modulation is a viable therapeutic strategy to delay T2DM-NAFLD progression.

## 1. Introduction

Non-alcoholic fatty liver disease (NAFLD) is a clinical pathological syndrome characterized by excessive hepatic lipid accumulation, which was primarily induced by metabolic risk factors, such as high dietary fat intake and obesity, that are distinct from other established etiologies, including alcohol consumption, hepatotoxic medications, and genetic disorders [1]. Globally, NAFLD represents a highly prevalent chronic liver condition. According to histopathological severity, characterized by inflammatory activity and fibrotic burden, NAFLD is classified into distinct stages: non-alcoholic fatty liver (NAFL) or simple steatosis, non-alcoholic steatohepatitis (NASH), progressive hepatic fibrosis (HF), cirrhosis, and hepatocellular carcinoma (HCC) [2]. The global prevalence of NAFLD increased from 25.26% (21.59–29.33) in 1990–2006 to 38.00% (33.71–42.49) in 2016–2019 [3]. Recent clinical investigations have indicated that NAFLD patients concurrently diagnosed with type 2 diabetes mellitus (T2DM) exhibited an elevated risk of developing advanced HF, cirrhosis, and HCC [4]. Numerous reports have shown a complex bidirectional relationship between NAFLD and T2DM [5]. Specifically, NAFLD patients who exhibit insulin resistance frequently possess a heightened susceptibility to impaired fasting glucose, which is a precursor to overt diabetes. Conversely, the majority of T2DM patients develop NAFLD manifestations, ranging from NAFL and NASH to more severe hepatic sequelae, including cirrhosis and HCC, with the concomitant acceleration of liver disease progression [6,7].

A recent study published in the Journal of Hepatology, a leading international hepatology journal, demonstrated that substantial free fatty acid overflow resulting from the disrupted metabolic environment during insulin resistance may interact with local hepatic mediators of inflammation, potentially underpinning the pathogenesis and development of NAFLD [8]. Under conditions of insulin resistance, impaired glucose utilization promotes lipolysis. The liberated free fatty acids predominantly undergo oxidative metabolism within hepatocytes. Ketone bodies, which are intermediates of fatty acid oxidation, comprise acetoacetate (AcAc), β-hydroxybutyrate (BHB), and trace acetone. Mitochondrial acetyl-CoA and acetoacetyl-CoA undergo condensation catalyzed by hydroxymethylglutaryl-CoA synthase 2 (HMGCS2; the rate-limiting ketogenic enzyme), forming hydroxymethylglutaryl-CoA (HMG-CoA), which is subsequently cleaved by HMG-CoA lyase to yield acetoacetate. The majority of AcAc is then reduced to BHB via β-hydroxybutyrate dehydrogenase [9]. Although hepatocytes possess a highly active ketogenic system, they lack the enzymatic machinery for ketone body utilization. Under physiological conditions, hepatocyte-derived ketone bodies enter systemic circulation and are transported to extrahepatic tissues. Within mitochondrial matrices, they are reconverted to acetyl-CoA for oxidation via the tricarboxylic acid cycle, thereby serving as a critical alternative energy substrate during states of carbohydrate insufficiency (e.g., fasting, prolonged exercise). Elevated serum ketone body concentrations have been documented in subsets of NAFL patients, with evidence suggesting that this metabolic alteration may correlate with increased mortality in the later stages [10]. Conversely, as NAFL progresses to NASH and HF, ketogenesis becomes impaired, leading to significantly reduced ketone body levels [11,12]. These observations indicate that ketone body dysregulation may significantly contribute to disease pathogenesis, although the precise mechanistic basis for this metabolic shift remains incompletely characterized.

The primary component of ketone bodies, BHB, functions as an alternative energy source and a metabolic signaling mediator that enhances antioxidant activity, anti-inflammatory effects, and anti-aging mechanisms [13]. Studies revealed that ketogenesis, a process that involves HMGCS2, provides critical energy substrates to decelerate aging during cellular senescence [14]. A study in the Journal of Hepatology revealed significantly increased hepatocyte senescence during progression from NAFL to NASH and HF [15]. Subsequent research in Nature Communications demonstrated that hepatocyte senescence in NAFLD promotes the release of inflammatory factors and exacerbates hepatic steatosis [16]. Consequently, the role of HMGCS2-regulated ketogenesis in modulating hepatocyte senescence during NAFL-to-NASH progression in T2DM warrants further investigation.

Cynaroside, also known as luteoloside, is a flavonoid compound that is abundant in Apiaceae, Poaceae, Lamiaceae, Solanaceae, Zingiberaceae, Asteraceae, and other plant families. It exhibits diverse pharmacological activities, including antibacterial, antifungal, antileishmanial, antioxidant, hepatoprotective, antidiabetic, anti-inflammatory, and anticancer properties [17]. Current research on cynaroside in fatty liver disease remains limited, with a recent comprehensive investigation employing a palmitic acid (PA)-induced in vitro NAFLD model [18]. No studies have specifically examined NAFLD that is comorbid with T2DM. This study will therefore investigate whether dysregulated ketogenesis correlates with the pathophysiological progression of NAFLD in the context of T2DM. Furthermore, we will elucidate the therapeutic potential of cynaroside in ameliorating NASH with T2DM through the regulation of HMGCS2-dependent ketogenesis and explore its underlying molecular mechanisms.

## 2. Materials and Methods

### 2.1. Reagents and Antibodies

Cynaroside (Herbpurify, Chengdu, China); streptozotocin (STZ, MedChemExpress, Monmouth Junction, NJ, USA); an alanine aminotransferase (ALT), aspartate aminotransferase (AST), total cholesterol (TC), and triglyceride (TG) detection kit (Nanjing Jiancheng Bioengineering Institute, Nanjing, China); an acetoacetic acid (AcAc) detection kit (BC5075, Solarbio, Beijing, China); a β-hydroxybutyric acid (BHB) detection kit (BC5085, Solarbio, Beijing, China); an ECL detection kit (Millipore, WBKLS0500, Burlington, MA, USA); an Oil Red O Staining Kit (Cell Signaling Technology, Danvers, MA, USA); hematoxylin and eosin (H&E) staining solution (Leagene Bio, Beijing, China); an SA-β-gal staining kit (Cell Signaling Technology, Danvers, MA, USA); a BCA protein concentration assay kit (Beyotime, Shanghai, China); HMGCS2 (Cell Signaling Technology, Danvers, MA, USA); p53, p21, and β-actin (Proteintech Group, Chicago, IL, USA); and goat anti-mouse IgG antibody [HRP], goat anti-rabbit IgG antibody [HRP], CoraLite594 labeled goat anti-mouse IgG antibody, and CoraLite488 labeled goat anti-rabbit IgG antibody (Proteintech Group, Chicago, IL, USA).

### 2.2. Experimental Animal Procedures

The animals (Henan Skbex Biotechnology Co., Ltd., Anyang, China) were maintained under standard laboratory conditions at the Department of Pharmacology, Wannan Medical College, China. These conditions included a temperature of 22 ± 2 °C, a relative humidity of 51 ± 5%, and a 12 h light–dark cycle. Mice were provided ad libitum access to food and water, except during experimentally mandated fasting periods. The animal study protocol was approved by the Institutional Animal Care and Use Committee of Wannan Medical College (Approval No. WNMC-AME-2024411).

#### 2.2.1. Experimental Group Assignment, Maintenance, and Therapeutic Regimen for the First Batch of C57BL/6J Mice

Following a 1-week acclimatization period, 4-week-old male C57BL/6J mice were randomly assigned to two experimental groups: the CDAHFD group (*n* = 30) and the STZ + CDAHFD group (*n* = 30). Both groups received a methionine–choline-deficient, high-fat diet supplemented with 1% cholesterol (CDAHFD; 45% kcal from fat). Additionally, mice in the STZ + CDAHFD group received intraperitoneal injections of STZ (60 mg/kg body weight) for three consecutive days. These injections were administered at weeks 0, 2, 4, 6, and 8, following a 12 h fast initiated on the fifth day of each respective week. One week after injection at each time point, fasting blood glucose (FBG) levels were measured. Subsets of mice (*n* = 5 per group per time point) were humanely euthanized at the conclusion of weeks 0, 2, 4, 6, 8, and 10. Euthanasia was performed under sodium pentobarbital anesthesia. Blood samples were collected via the orbital sinus puncture method, and liver tissue specimens were harvested for subsequent analyses.

A separate cohort of 4-week-old male C57BL/6J mice underwent the same 1-week acclimatization. These mice were then maintained on a standard rodent growth diet. Subsets of mice (*n* = 3) were similarly euthanized and sampled (blood via orbital puncture, liver tissue collection) at weeks 0, 2, 4, 6, 8, and 10 for comparative analysis.

#### 2.2.2. Experimental Group Assignment, Maintenance, and Therapeutic Regimen for the Second Batch of C57BL/6J Mice

Four-week-old male C57BL/6J mice (*n* = 32) were randomly divided into four groups (*n* = 8) after one week of adaptive feeding: 1. Control group: given normal maintenance chow for 8 weeks, and at week 5, the appropriate amount of saline was injected intraperitoneally for three consecutive days after 12 h of fasting; 2. Empty vector group: normal maintenance feed for 8 weeks, and at week 5, an appropriate amount of saline was injected intraperitoneally for three days after 12 h of fasting, along with tail vein injection of AAV8 adenovirus-packed empty vector (Luciferase labeling); 3. Model + empty vector group: CDAHFD for 8 weeks, and at week 5, STZ 60 mg/kg was injected intraperitoneally for three consecutive days after 12 h of fasting, along with AAV8 adenovirus-packed empty vector (Luciferase labeling) injected into the tail vein; and 4. Model + HMGCS2 overexpression group: CDAHFD was administered for 8 weeks, and at week 5, STZ 60 mg/kg was injected intraperitoneally for three consecutive days after 12 h of fasting, while AAV8 adenovirus-packaged HMGCS2 overexpression plasmid (Luciferase labeling) was injected into the tail vein. The successful induction of type 2 diabetes was confirmed by FBG levels exceeding 11.1 mmol/L in NASH + T2DM mice.

#### 2.2.3. Experimental Group Assignment, Maintenance, and Therapeutic Regimen for the Third Batch of C57BL/6J Mice

A total of 40 male C57BL/6J mice (4 weeks old) were randomly divided into five groups (*n* = 8) after 1 week of adaptive feeding: 1. Control group: The control group received standard chow for 8 weeks. At week 5, vehicle control (saline) was administered intraperitoneally for three consecutive days following a 12 h fast, with daily saline gavage during weeks 5–8. 2. Model group: This group was maintained on CDAHFD for 8 weeks. Following a 12 h fast at week 5, STZ (60 mg/kg/day) was intraperitoneally administered for three consecutive days. Concurrently, daily saline gavage was maintained during weeks 5–8. 3. Cynaroside (12.5 mg/kg) group: This group was fed CDAHFD for 8 weeks. STZ (60 mg/kg) was administered intraperitoneally for three days at week 5 post-fasting, with concurrent daily oral gavage of cynaroside (12.5 mg/kg) from week 5 to week 8. 4. Cynaroside (25 mg/kg) group: CDAHFD was given for 8 weeks. STZ (60 mg/kg) was injected intraperitoneally for three consecutive days at week 5 following fasting, supplemented with daily cynaroside (25 mg/kg) via gavage during weeks 5–8. 5. Cynaroside (50 mg/kg) group: CDAHFD was given for 8 weeks. STZ (60 mg/kg) was delivered intraperitoneally for three days at week 5 after fasting, co-administered with daily cynaroside (50 mg/kg) via gavage throughout weeks 5–8. During this experimental procedure, one mouse per group in both the medium- and high-dose cynaroside groups died due to improper performance of the gavage technique.

#### 2.2.4. Experimental Group Assignment, Maintenance, and Therapeutic Regimen for the Fourth Batch of C57BL/6J Mice

Following a 1-week acclimatization period, 40 male C57BL/6J mice (4 weeks old) were randomly allocated to five experimental groups (*n* = 8 per group): 1. Control group: The control group received normal maintenance chow for 8 weeks. At week 5, vehicle solution (saline) was administered intraperitoneally for three consecutive days following a 12 h fast, with daily saline gavage throughout weeks 5–8. 2. sh-NC group: This group was given normal maintenance feed for 8 weeks. At week 5, saline was injected intraperitoneally for three consecutive days post-12 h fast, accompanied by daily saline gavage during weeks 5–8. Concurrently, AAV8 adenovirus-packaged scrambled shRNA (scr shRNA, Luciferase labeling) was delivered via tail vein injection. 3. CDAHFD + STZ + sh-NC group: CDAHFD was fed to the mice for 8 weeks. At week 5, streptozotocin (60 mg/kg) was administered intraperitoneally for three consecutive days after a 12 h fast, with daily saline gavage during weeks 5–8. scr shRNA adenovirus was co-administered via tail vein injection; 4. CDAHFD + STZ + sh-NC + cynaroside (25 mg/kg) group: CDAHFD was administered for 8 weeks. Streptozotocin (60 mg/kg) was injected intraperitoneally for three consecutive days at week 5 post-fasting. Daily oral cynaroside (25 mg/kg) and scr shRNA adenovirus were administered via gavage and tail vein injection, respectively, throughout weeks 5–8. 5. CDAHFD + STZ + HMGCS2 shRNA + cynaroside (25 mg/kg) group: CDAHFD was given for 8 weeks. Streptozotocin (60 mg/kg) was delivered intraperitoneally for three days at week 5 after fasting. Daily interventions included cynaroside (25 mg/kg) administered via gavage and AAV8 adenovirus-packaged HMGCS2 shRNA (Luciferase labeling) administered via tail vein injection during weeks 5–8.

At the terminal endpoint, mice were anesthetized with sodium pentobarbital. Blood samples were collected via the orbital sinus puncture method, and hepatic tissues were harvested for subsequent analyses.

### 2.3. Serum Biochemical Analysis

Blood samples were collected using vacuum blood collection tubes (containing separating gel and procoagulant) (Jiangsu Yuli Medical Instrument Co., Ltd., Taizhou, China), left for 1 h, and centrifuged at 4 °C and 3500 rpm for 15 min. The upper serum layer was transferred and stored at −80 °C. ALT, AST, TC, TG, AcAc, and BHB were detected using commercial assay kits according to the manufacturer’s protocol.

### 2.4. H&E Staining

Fresh hepatic tissue specimens were fixed in 10% neutral buffered formalin for 24–48 h, subsequently dehydrated, cleared, and embedded in paraffin blocks. Serial sections of 4 μm thickness were cut using a microtome, deparaffinized in xylene, and rehydrated through a graded ethanol series. Sections were then routinely stained with H&E according to standard histological protocols. Image J(V1.8.0) software (NIH, Bethesda, MD, USA) was used to quantify the steatosis area, as well as the inflammation area on the micrographs.

### 2.5. Oil Red O Staining

Oil Red O staining was employed to visualize lipid droplets. Liver tissues were embedded in Optimal Cutting Temperature (OCT) compound and flash-frozen in liquid nitrogen-cooled isopentane. Cryosections 8 μm thick were prepared using a cryostat (CryoStar NX50 OPD, Thermo Fisher Scientific, Waltham, MA, USA) maintained at −20 °C. The Oil Red O working solution was filtered through a 0.22 μm syringe filter (Millex^TM^-GP, Millipore, Burlington, MA, USA) immediately before use. Subsequent processing steps, including counterstaining and mounting, were performed in strict accordance with the manufacturer’s protocol. Image J software was used to quantify the Oil Red O area on the micrographs.

### 2.6. SA-β-Gal Staining

SA-β-gal histochemistry was employed to detect cellular senescence. Liver tissues were embedded in OCT compound, snap-frozen in liquid nitrogen-cooled isopentane, and sectioned at a thickness of 8 μm using a cryostat maintained at −20 °C. The SA-β-gal staining solution pH was adjusted to 5.9–6.1 with 0.1 M citric acid/sodium phosphate buffer. Subsequent procedures were performed according to the manufacturer’s protocol. The total cell numbers and stained cell numbers were counted from 3 fields per sample. SA-β-gal-positive cells were calculated as the percentage of positive cells per unit area.

### 2.7. Western Blot Assay

Mouse hepatic tissues were homogenized in ice-cold RIPA lysis buffer (containing 1% protease inhibitor PMSF) for 30 min. The supernatant was aspirated after centrifugation at 12,000 rpm for 15 min at 4 °C, and the protein concentration was measured using a BCA kit. Protein lysates were resolved via SDS-PAGE (10–12% gels) and electrophoretically transferred onto PVDF membranes. After blocking with 5% non-fat dry milk in TBST for 2 h at room temperature, membranes were incubated with primary antibodies (dilution 1:1000) overnight at 4 °C. Following three 10 min TBST washes, membranes were probed with horseradish peroxidase (HRP)-conjugated secondary antibodies (1:5000 dilution) for 2 h at room temperature. Protein signals were detected using an enhanced chemiluminescence (ECL) kit (Millipore, WBKLS0500, Burlington, MA, USA) and visualized on a ChemiDoc imaging system. Band intensities were quantified using Image Lab Software (Amersham ImageQuant 800, Cytiva, Uppsala, Sweden) or Image J software (NIH, Bethesda, MD, USA) and normalized to a β-actin loading control.

### 2.8. FBG Detection

Following a 12 h fasting period (ad libitum water access), mice were administered STZ (60 mg/kg body weight) via intraperitoneal injection for three consecutive days. The compound was dissolved in 0.1 mol/L citrate buffer (pH 4.5), and each dose was delivered within 30 min of preparation. Blood glucose levels were quantified from tail vein samples using enzymatic test strips. Successful induction of type 2 diabetes was confirmed by FBG levels exceeding 11.1 mmol/L.

### 2.9. Determination of Reactive Oxygen Species (ROS)

ROS were measured using the ROS Assay Kit (Beyotime, Shanghai, China) according to the manufacturer’s instructions. Briefly, frozen liver sections (4 μm) were incubated with 20 μM dihydroethidium (DHE) in the dark at 37 °C for 30 min and then counterstained with antifade mounting medium. Slides were mounted and observed under a laser confocal microscope.

### 2.10. Detection of Serum Inflammatory Cytokines

Serum samples, which were obtained following final drug administration and separated via centrifugation, were assayed to quantify concentrations of inflammatory cytokine tumor necrosis factor-α (TNF-α) and interleukin-1 beta (IL-1β), employing enzyme-linked immunosorbent assay (ELISA) kits (Jiangsu Meimian Industrial Co., Ltd., Yancheng, China). All protocols were executed in strict accordance with the manufacturer’s specifications. After terminating the reactions, optical density (OD) at 450 nm was determined using a microplate reader. Calibration curves were established using OD values from kit-supplied reference standards of known concentrations. Thereafter, target cytokine concentrations in all serum specimens were derived for each experimental cohort.

### 2.11. Proteomic Profiling

Samples were homogenized in protein lysis buffer containing protease inhibitors using a high-flux tissue grinder (3 cycles, 40 s each). The lysate was incubated on ice for 30 min with intermittent vortexing (5–10 s every 5 min). Following centrifugation (16,000× *g*, 4 °C, 30 min), supernatant protein concentration was determined using a Bicinchoninic acid assay kit (Thermo Scientific, Waltham, MA, USA). Aliquots (100 µg protein) were resuspended in 100 mM triethylammonium bicarbonate (TEAB) buffer. Proteins were reduced with 10 mM tris (2-carboxyethyl) phosphine (TCEP; 37 °C, 60 min) and alkylated with 40 mM iodoacetamide (IAM; room temperature, 40 min, dark). After centrifugation (10,000× *g*, 4 °C, 20 min), the pellet was collected, resuspended in 100 µL 100 mM TEAB, and digested with trypsin (1:50 *w*/*w*) at 37 °C overnight. Digested peptides were vacuum-dried, resolubilized in 0.1% trifluoroacetic acid, desalted using HLB cartridges, and vacuum-concentrated. Peptide concentration was quantified via UV absorbance using a NanoDrop One spectrophotometer (Thermo Scientific, Waltham, MA, USA). Quantified peptides were analyzed via a Vanquish Neo UHPLC system coupled to an Orbitrap Astral mass spectrometer (Thermo Scientific) at Majorbio Bio-Pharm. DIA was performed over *m*/*z* ranges of 70–1050 (MS1) and 150–2000 (MS2). Proteomic data analysis was performed using the Majorbio Cloud Platform. DEPs were defined by |fold change| > 1.2 and *p*-value < 0.05, determined using Student’s *t*-test in R programming language (University of Auckland, Oakland, NJ, USA).

### 2.12. Statistical Analysis

The results are presented as mean ± SD, and the differences between groups were analyzed using GraphPad Prism 7.0 (GraphPad Software, San Diego, CA, USA). The significance of the difference was determined via a one-way analysis of variance with the post hoc Dunnett’s test. Values of *p* < 0.05 were considered statistically significant.

## 3. Results

### 3.1. Impaired Ketogenesis May Be Associated with Accelerated Hepatic Injury, Dyslipidemia, and Cellular Senescence During the Progression from NAFL to NASH in Individuals with T2DM

**Figure 1:**
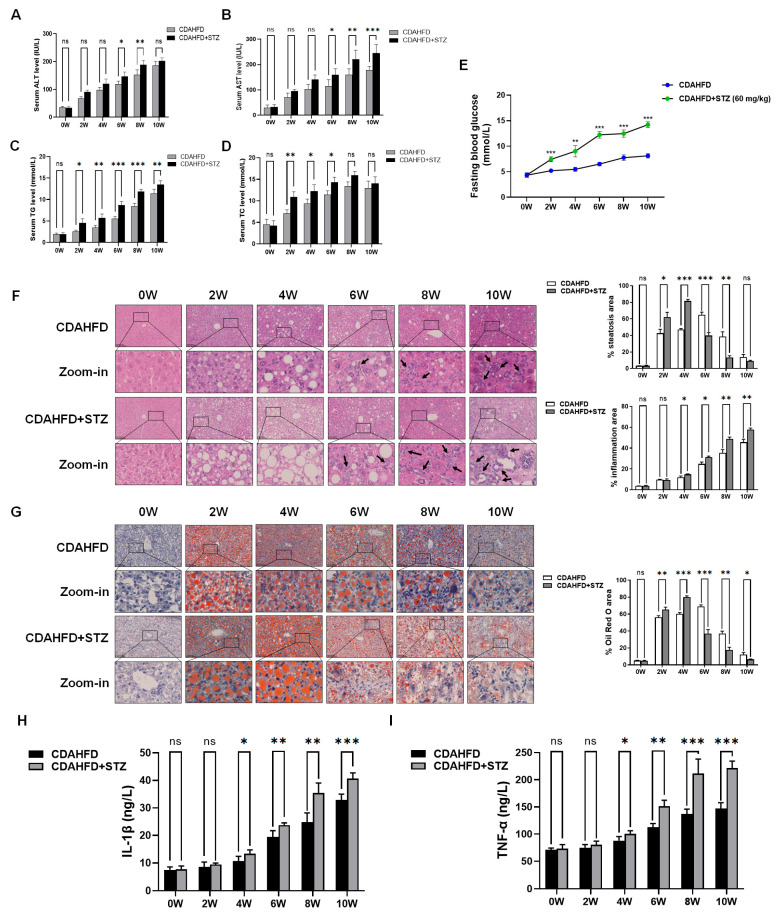
Liver injury and dyslipidemia worsen during the progression from NAFL to NASH in individuals with T2DM. (**A**–**D**) Serum levels of liver injury markers (ALT, AST) and lipid profiles (TG, TC) were measured (*n* = 3). Significance: * *p* < 0.05, ** *p* < 0.01, *** *p* < 0.001. (**E**) Blood glucose meter was used to detect fasting blood glucose in mice (*n* = 5). Significance: ** *p* < 0.01, *** *p* < 0.001 vs. CDAHFD group. (**F**) Histopathological alterations in hepatocyte lipid accumulation and inflammatory infiltration during the progression from NAFL to NASH in individuals with T2DM (*n* = 3). Representative H&E-stained liver sections. Scale bar = 100 µm. Inflammation is marked with black arrows. Bar graphs showed the percentage quantification of steatosis and inflammation area using Image J software, as shown in Figure 1F. Significance: * *p* < 0.05, ** *p* < 0.01, *** *p* < 0.001. (**G**) Progressive changes in hepatocyte lipid accumulation during the transition from NAFL to NASH in T2DM. Representative Oil Red O-stained liver sections (*n* = 3). Scale bar = 100 µm. Bar graphs showed the percentage quantification of Oil Red O area using Image J software, as shown in Figure 1G. Significance: * *p* < 0.05, ** *p* < 0.01, *** *p* < 0.001. (**H**,**I**) The levels of the inflammatory genes IL-1β and TNF-α in the serum of C57BL/6J mice were detected using ELISA kits. Significance: * *p* < 0.05, ** *p* < 0.01, *** *p* < 0.001.

**Figure 2:**
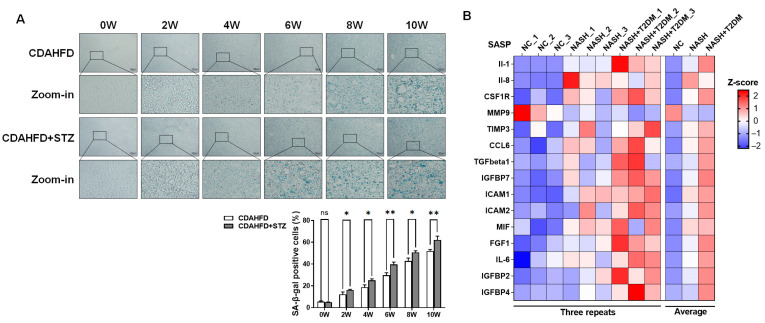
Hepatocyte senescence progressively increased during the transition from NAFL to NASH, with significant exacerbation observed in T2DM-NAFLD cohorts. (**A**) Representative SA-β-gal-stained liver sections (*n* = 3). Scale bar = 100 µm. Significance: * *p* < 0.05, ** *p* < 0.01. (**B**) Proteomic analysis was performed to quantify the expression of pro-inflammatory SASP factors in hepatic tissue from control, NASH, and NASH-T2DM comorbidity mice models (*n* = 3).

To investigate the degree of liver damage in the progression of NAFLD with T2DM, we established a murine model by administering CDAHFD and STZ to C57BL/6J mice for 2–10 weeks, simulating the transition from NAFL to NASH with T2DM. Serological analyses revealed progressively upregulated hepatic injury markers (ALT, AST) during the progression of NAFLD. Notably, the NAFLD-T2DM cohort exhibited significantly greater injury than NAFLD-only controls (Figure 1A,B). Concurrently, lipid profiles (TG, TC) increased throughout NAFLD modeling, with T2DM comorbidity exacerbating dyslipidemia (Figure 1C,D). Blood glucose analysis revealed that mice subjected to combined STZ and CDAHFD induction developed hyperglycemia (exceeding 6.1 mmol/L) by week 2, which was markedly elevated compared to those receiving CDAHFD alone. Critically, fasting blood glucose concentrations surpassed the diagnostic threshold for T2DM (≥11.1 mmol/L) after 6 weeks of concurrent CDAHFD and STZ administration, exhibiting a time-dependent progressive elevation throughout the intervention period. These findings confirmed the successful establishment of a T2DM model through 6-week CDAHFD feeding coupled with triple low-dose STZ injections (Figure 1E). H&E and Oil Red O staining demonstrated time-dependent enhancment in hepatocellular disorganization, lobular architecture loss, and lipid accumulation during the NAFL modeling period (weeks 2–4), with those in T2DM-NAFLD specimens markedly intensified. By week 6, hepatic lipid vacuoles continued to increase in the CDAHFD group. In contrast, mice receiving both CDAHFD and STZ exhibited a significant reduction in hepatic lipid vacuoles, concomitant with progressively expanding areas of inflammatory infiltration (Figure 1F,G). Additionally, we measured the serum levels of inflammatory markers TNF-α and IL-1β. We found that in the CDAHFD model group, these markers showed no significant changes at either 2 or 4 weeks. However, their expression began to increase significantly starting at week 6. In contrast, mice subjected to the combined CDAHFD + STZ modeling exhibited a significant increasing trend in serum TNF-α and IL-1β levels as early as week 4, indicating that STZ combined with CDAHFD accelerated the progression from NAFL to NASH (Figure 1H,I). Therefore, based on the experimental findings described above, we used 8-week CDAHFD plus STZ treatment in mice to establish a NASH combined with T2DM model in subsequent experiments. The analysis of SA-β-gal staining showed progressive hepatocyte senescence during NAFLD progression, which was significantly amplified in T2DM-NAFLD groups (Figure 2A). Proteomics identified upregulated proinflammatory senescence-associated secretory phenotype (SASP) factors in NASH mice. Remarkably, the livers NASH + T2DM mice exhibited further SASP expansion, suggesting T2DM accelerated hepatocyte senescence in NASH (Figure 2B).

**Figure 3:**
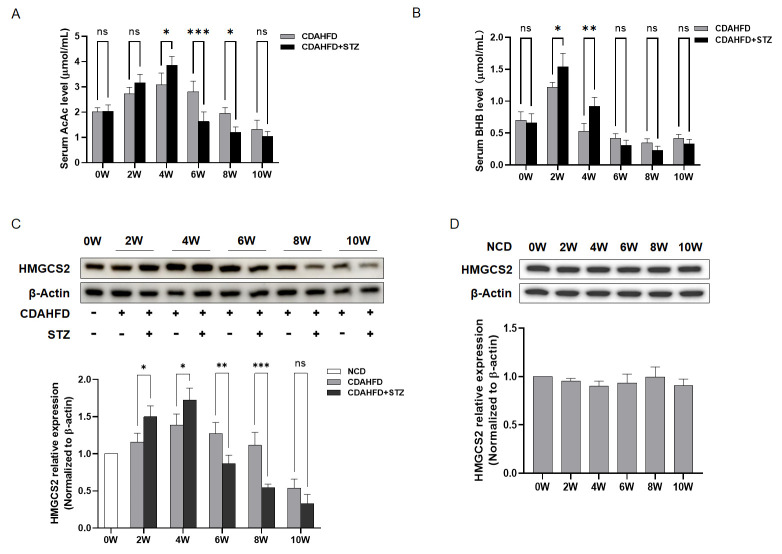
Ketone body levels became dysregulated during the progression from NAFL with T2DM to NASH, suggesting a potential association with HMGCS2 regulation. (**A**,**B**) Serum concentrations of ketone bodies AcAc and BHB were measured (*n* = 3). Significance: * *p* < 0.05, ** *p* < 0.01, *** *p* < 0.001. (**C**,**D**) Representative western blot analysis and quantification of hepatic HMGCS2 protein expression (*n* = 3). Significance: * *p* < 0.05, ** *p* < 0.01, *** *p* < 0.001.

Ketone body homeostasis was investigated through the quantitative measurement of serum AcAc and BHB concentrations to elucidate its role in NAFLD progression with T2DM comorbidity. Experimental results demonstrated dynamic alterations in ketone metabolism during the progression of fatty liver disease. Serum AcAc levels increased significantly during weeks 0–4, while BHB elevation was observed at weeks 0–2. Concomitant T2DM amplified these ketogenic responses. However, as the disease advanced to steatohepatitis, ketone body concentrations declined substantially, with T2DM comorbidity exacerbating this reduction (Figure 3A,B). Analysis of the rate-limiting ketogenic enzyme HMGCS2 revealed significantly elevated expression in T2DM-NAFLD cohorts compared to NAFLD controls during the early stages of the disease (weeks 0–4). Conversely, extending the modeling to 10 weeks induced progressive HMGCS2 downregulation, which was markedly intensified by T2DM comorbidity (Figure 3C). Furthermore, no significant correlation was found between murine age and HMGCS2 expression (Figure 3D), indicating that the changes in HMGCS2 expression were disease phase-dependent rather than age-dependent.

### 3.2. Impaired Ketogenesis Observed in NASH Complicated by T2DM May Be Mechanistically Linked to the Dysregulation of HMGCS2

**Figure 4:**
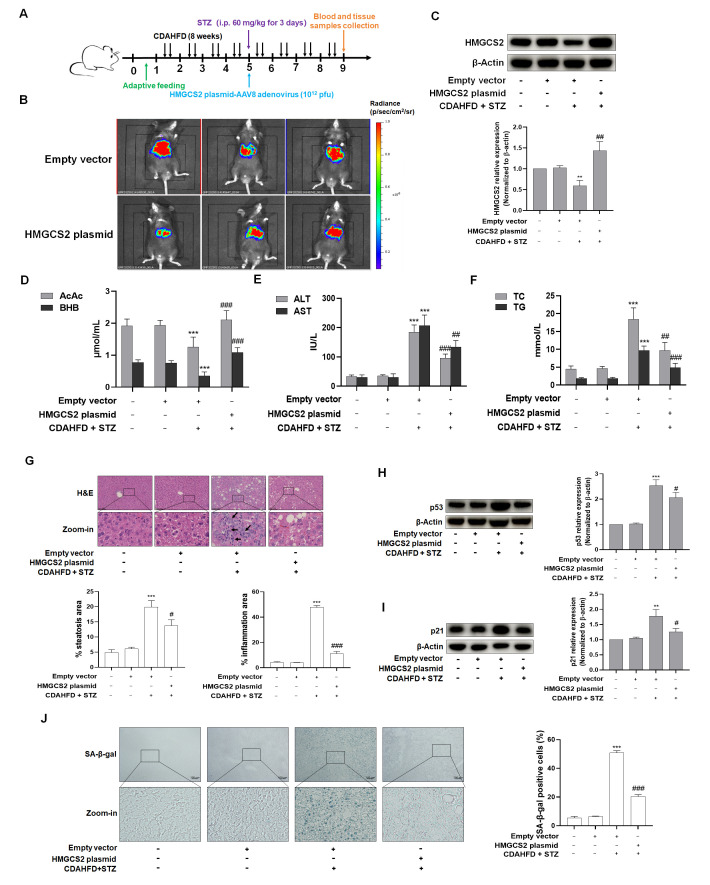
Hepatocyte-specific HMGCS2 overexpression ameliorated NASH with concomitant T2DM in C57BL/6J mice via enhanced ketogenesis. (**A**) Experimental setup of mice. AAV8 adenovirus delivery: HMGCS2 plasmid and AAV8 adenovirus scramble were administered to CDAHFD + STZ-treated mice. Intraperitoneal injection (i.p.). (**B**) Bioluminescence imaging using the Caliper IVIS Lumina II system confirmed the predominant hepatic localization of HMGCS2. (**C**) Western blot analysis confirming HMGCS2 plasmid transfection efficiency in the liver (*n* = 3). Significance: ** *p* < 0.01 vs. Empty vector (EV); ^##^ *p* < 0.01 vs. EV-CDAHFD-STZ (60 mg/kg) group. (**D**) Serum concentrations of ketone bodies AcAc and BHB were measured (*n* = 6). Significance: *** *p* < 0.001 vs. EV; ^###^ *p* < 0.001 vs. EV-CDAHFD-STZ group. (**E**,**F**) Serum levels of hepatic injury markers (ALT, AST) and lipid profiles (TG, TC) were measured (*n* = 5). Significance: *** *p* < 0.001 vs. EV; ^##^ *p* < 0.01, ^###^ *p* < 0.001 vs. EV-CDAHFD-STZ group. (**G**) Inflammatory infiltration and lipid accumulation in liver sections were detected using H&E-staining (*n* = 3). Representative images of H&E-stained liver sections are shown. Scale bar = 100 µm. Inflammation is marked with black arrows. Bar graphs show the percentage quantification of steatosis and inflammation areas using Image J software, as shown in Figure 4G. Significance: *** *p* < 0.001 vs. EV group; ^#^ *p* < 0.05, ^###^ *p* < 0.001 vs. EV-CDAHFD-STZ (60 mg/kg) group. (**H**,**I**) Quantitative analysis of hepatic senescence markers p53 and p21 protein expression via western blot (*n* = 3). Significance: ** *p* < 0.01, *** *p* < 0.001 vs. EV; ^#^ *p* < 0.05 vs. EV-CDAHFD-STZ group. (**J**) SA-β-gal staining revealed hepatocyte senescence in liver sections of C57BL/6J mice (*n* = 3). Representative images shown. Scale bar = 100 µm. Significance: *** *p* < 0.001 vs. EV group; ^###^ *p* < 0.001 vs. EV-CDAHFD-STZ (60 mg/kg) group.

To explore whether impaired ketogenesis in NASH with T2DM stems from dysregulated HMGCS2, C57BL/6J mice were treated with CDAHFD and STZ for 8 weeks to establish a NASH combined with T2DM model. Subsequently, HMGCS2 overexpression was induced via the tail vein injection of AAV8 adenovirus-packaged HMGCS2 overexpression plasmid (Figure 4A). In vivo bioluminescence imaging confirmed the predominant hepatic localization of HMGCS2 (Figure 4B), while Western blot analysis verified successful transfection efficiency (Figure 4C). Furthermore, we assessed the transfection efficiency of the HMGCS2-overexpressing plasmid in cardiac and renal tissues. The results demonstrated that cardiac and renal HMGCS2 expression showed no significant increase in the overexpression cohort relative to the model group controls. This indicated that following tail vein injection, the AAV8-packaged HMGCS2-overexpressing plasmid exhibited primary liver-specific tropism (Figure S1). Serum levels of AcAc and BHB were significantly reduced in the NASH + T2DM model group relative to the controls. Conversely, HMGCS2 overexpression substantially elevated both ketone bodies compared to the model group, indicating enhanced ketogenesis in NASH + T2DM mice (Figure 4D). This result suggested that abnormal ketone body production in the NASH with T2DM model may be associated with the dysregulation of HMGCS2. Furthermore, hepatic injury markers (ALT, AST) and lipid metabolism indicators (TC, TG) were markedly increased in the model group versus the controls. These parameters were significantly attenuated following HMGCS2 overexpression (Figure 4E,F). Histopathological assessment revealed substantial hepatic steatosis and inflammatory infiltration in NASH + T2DM mice. These pathological features were significantly attenuated in HMGCS2-overexpressing mice compared to vector controls (Figure 4G). Furthermore, Western blot analysis showed enhanced expression of senescence-associated proteins p53 and p21, and SA-β-gal staining demonstrated increased senescent cell prevalence in NASH + T2DM livers. Both parameters were markedly reduced following HMGCS2 overexpression (Figure 4H–J). These findings suggest that HMGCS2 plays an important role in the progression of NAFLD and might be used as a therapeutic target for delaying NAFLD-T2DM progression.

### 3.3. Cynaroside Improves NASH Combined with T2DM by Regulating Ketogenesis

**Figure 5:**
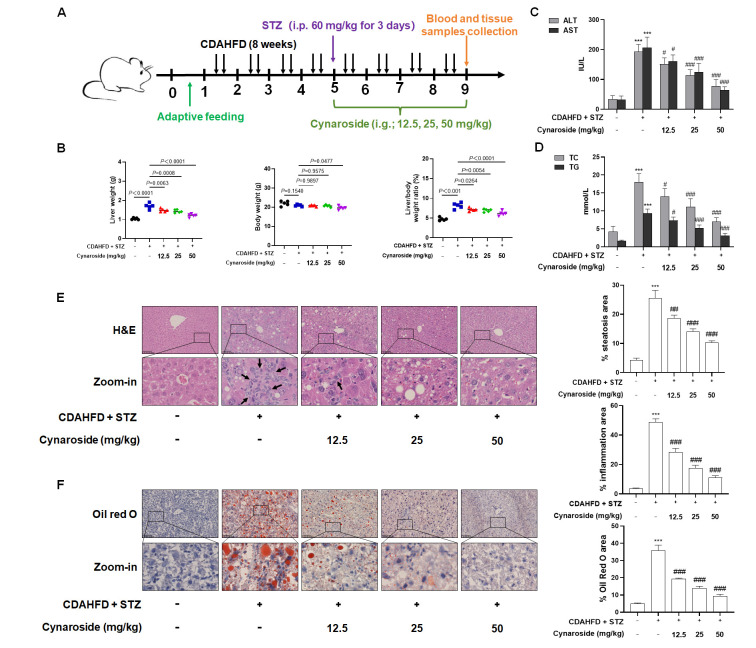
Cynaroside ameliorated liver injury and dyslipidemia in NASH with concomitant T2DM mice. (**A**) Experimental setup of mice. Intragastric administration (i.g.). (**B**) Determination of the weight of the liver and body, as well as the liver-to-body weight ratio. (**C**,**D**) Determination of serum levels of liver injury markers (ALT, AST) and lipid profiles (TG, TC) (*n* = 5); *** *p* < 0.001 vs. NC group; *^#^ p* < 0.05, *^##^ p* < 0.01, *^###^ p* < 0.001 vs. CDAHFD + STZ (60 mg/kg) group. (**E**,**F**) Representative images of H&E and Oil Red O-stained liver sections demonstrating inflammatory infiltration and lipid accumulation (*n* = 3). Scale bar = 100 µm. Inflammation is marked with black arrows. Bar graphs show the percentage quantification of steatosis, inflammation, and the Oil Red O area using Image J software, as shown in Figure 5E,F. Significance: *** *p* < 0.001 vs. NC group; *^##^ p* < 0.01, *^###^ p* < 0.001 vs. CDAHFD + STZ (60 mg/kg) group.

To evaluate the therapeutic efficacy of cynaroside in C57BL/6J mice with CDAHFD- and STZ-induced NASH concomitant with T2DM, model animals were administered varying doses of cynaroside (Figure 5A). Compared with the controls, both absolute liver weight and relative liver weight (liver-to-body weight ratio) were significantly increased in the model group. Treatment with 12.5 mg/kg cynaroside attenuated these elevations, while 50 mg/kg cynaroside demonstrated a more evident enhancement of this suppressive effect (Figure 5B). Furthermore, serum indexes of liver injury (AST, ALT) and lipid metabolism (TC, TG) were significantly increased in NASH-T2DM mice. The administration of 12.5 mg/kg cynaroside ameliorated these abnormalities, with the 50 mg/kg dose producing significantly greater efficacy (Figure 5C,D). Histological analysis revealed substantial lipid accumulation and inflammatory infiltration in NASH-T2DM hepatic tissues compared to the controls, as demonstrated by H&E and Oil Red O staining (Figure 5E,F). These pathological features were markedly attenuated in cynaroside-treated groups, with the most pronounced improvement observed at the 50 mg/kg dose. Collectively, these results indicate that cynaroside improved STZ- and CDAHFD-induced hepatic injury and dyslipidemia in C57BL/6J mice.

**Figure 6:**
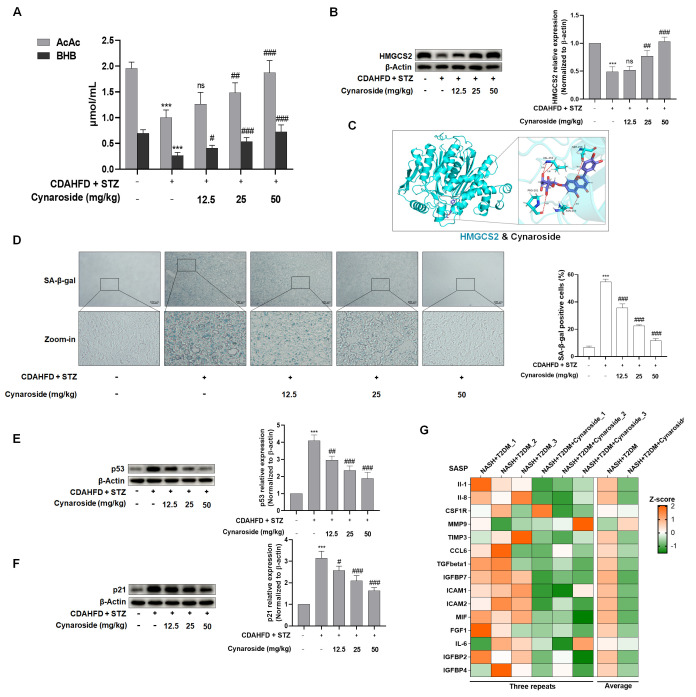
Cynaroside inhibited the cellular senescence of hepatocytes in mice with NASH C57BL/6J complicated by T2DM through the promotion of ketogenesis. (**A**) Determination of serum levels of ketone body markers (AcAc, BHB) (*n* = 5). Significance: *** *p* < 0.001 vs. NC group; ^#^ *p* <0.05, ^##^ *p* < 0.01, ^###^ *p* < 0.001 vs. CDAHFD + STZ (60 mg/kg) group. (**B**) Western blot analyses of HMGCS2 protein levels in liver tissues (*n* = 3). Significance: *** *p* < 0.001 vs. NC group; ^##^ *p* < 0.01, ^###^ *p* < 0.001 vs. CDAHFD + STZ (60 mg/kg) group. (**C**) Binding mode of cynaroside in the active site of HMGCS2. The 3D interaction pattern showed an expanded view of residues proximal to cynaroside within the binding site. (**D**) Representative images of liver sections stained with SA-β-gal staining (*n* = 3). Scale bar, 100 μm. Significance: *** *p* < 0.001 vs. NC group; ^###^ *p* < 0.001 vs. CDAHFD + STZ (60 mg/kg) group. (**E**,**F**) Western blot analyses of senescence proteins p53 and p21 protein levels in liver tissues (*n* = 3); *** *p* < 0.001 vs. NC group; ^#^*p* < 0.05, ^##^ *p* < 0.01, ^###^ *p* < 0.001 vs. CDAHFD + STZ (60 mg/kg) group. (**G**) Proteomic analysis was performed to quantify the expression of pro-inflammatory SASP factors in hepatic tissue from NASH + T2DM and NASH + T2DM + cynaroside mice models.

Furthermore, serum levels of ketone body-related indicators (AcAc, BHB) were quantified to assess cynaroside’s effects. The results demonstrated reduced ketone body concentrations in NASH-T2DM mice, whereas cynaroside administration significantly raised these levels (Figure 6A). Relative to the model group, HMGCS2 expression exhibited an upward trend in cynaroside-treated mice, with the 50 mg/kg dose producing a statistically significant enhancement (Figure 6B). To validate the interaction of cynaroside and HMGCS2, molecular docking was performed using an in silico homology model of HMGCS2. Computational analysis revealed that cynaroside binds within the ligand pocket, engaging PRO-201, VAL-253, ASN-204, and SER-258 residues, primarily through hydrogen bond formation (Figure 6C). Histological assessment revealed significantly increased cellular senescence in the hepatic tissues of the model group relative to controls. Cynaroside administration significantly attenuated this senescence phenotype, with maximal suppression observed at the 50 mg/kg dosage (Figure 6D–F). Proteomic profiling identified upregulated proinflammatory SASP factors in NASH+T2DM mice, which were suppressed by cynaroside intervention (Figure 6G). Furthermore, ROS levels were quantified to evaluate their association with hepatocyte senescence. Hepatic tissue analysis revealed substantially increased ROS levels in the CDAHFD+STZ group. Notably, cynaroside administration induced a reduction in ROS accumulation (Figure S2). Collectively, these findings suggest that cynaroside improved CDAHFD and STZ-induced hepatocyte senescence in NASH-T2DM mice, potentially by modulating HMGCS2 expression.

### 3.4. Cynaroside Alleviates NASH Combined with T2DM via HMGCS2-Mediated Ketogenesis

**Figure 7:**
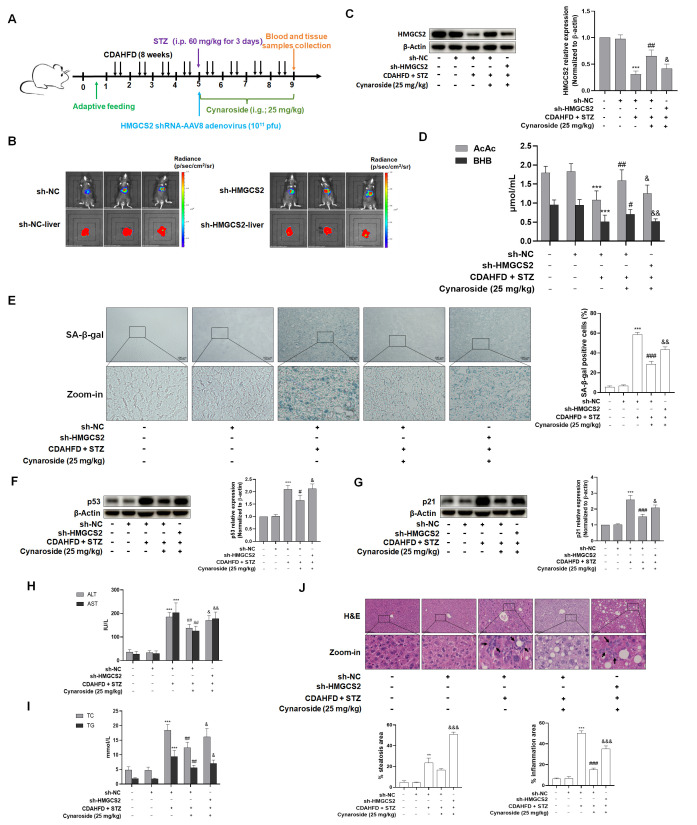
Down-regulation of HMGCS2 attenuated the ameliorative effect of cynaroside on NASH with T2DM by regulating hepatocyte senescence. (**A**) Experimental setup of mice. Intragastric administration (i.g.). Mice treated with CDAHFD+STZ were intravenously injected via the tail vein with AAV8 vectors delivering HMGCS2 shRNA and scramble shRNA. (**B**) Bioluminescence imaging using the Caliper IVIS Lumina II system confirmed the predominant hepatic localization of HMGCS2. (**C**) The transfection efficiency of HMGCS2 shRNA in the liver was evaluated via western blot analysis (*n* = 3). Significance: *** *p* < 0.001 vs. sh-NC group; ^##^ *p* < 0.01 vs. sh-NC + CDAHFD + STZ (60 mg/kg)-treated group; ^&^ *p* < 0.05 vs. sh-NC + CDAHFD + STZ (60 mg/kg) + cynaroside (25 mg/kg)-treated group. (**D**) Determination of serum levels of ketone body markers (AcAc, BHB) (*n* = 6). Significance: *** *p* < 0.001 vs. sh-NC group; ^#^ *p* < 0.05, ^##^ *p* < 0.01 vs. sh-NC + CDAHFD + STZ (60 mg/kg)-treated group; ^&^ *p* < 0.05, ^&&^ *p* < 0.01 vs. sh-NC + CDAHFD + STZ (60 mg/kg) + cynaroside (25 mg/kg)-treated group. (**E**) SA-β-gal staining revealed hepatocyte senescence in liver sections (*n* = 3). Representative images shown. Scale bar = 100 µm. Significance: *** *p* < 0.001 vs. sh-NC group; ^###^ *p* < 0.001 vs. sh-NC + CDAHFD + STZ (60 mg/kg)-treated group; ^&&^ *p* < 0.01 vs. sh-NC + CDAHFD + STZ (60 mg/kg) + cynaroside (25 mg/kg)-treated group. (**F**,**G**) Western blot analyses of p53 and p21 levels in liver tissues (*n* = 3). Significance: *** *p* < 0.001 vs. sh-NC group; ^#^ *p* < 0.05, ^###^ *p* < 0.001 vs. sh-NC + CDAHFD + STZ (60 mg/kg)-treated group; ^&^ *p* < 0.05 *vs.* sh-NC + CDAHFD + STZ (60 mg/kg) + cynaroside (25 mg/kg)-treated group. (**H**,**I**) Determination of serum levels of liver injury markers (ALT, AST) and lipid profiles (TG, TC) (*n* = 5). Significance: *** *p* <0.001 vs. sh-NC group; ^##^ *p* < 0.01 vs. sh-NC + CDAHFD + STZ (60 mg/kg) group; ^&^ *p* < 0.05, ^&&^ *p* < 0.01 vs. sh-NC + CDAHFD + STZ (60 mg/kg) + cynaroside (25 mg/kg)-treated group. (**J**) Representative images of liver sections stained with H&E staining (*n* = 3). Scale bar, 100 μm. Inflammation is marked with black arrows. Bar graphs show the percentage quantification of steatosis, and inflammation area using Image J software, as shown in Figure 7J. Significance: ** *p* < 0.01, *** *p* < 0.001 vs. sh-NC group; ^###^ *p* < 0.001 vs. sh-NC + CDAHFD + STZ (60 mg/kg)-treated group; ^&&&^ *p* < 0.001 vs. sh-NC + CDAHFD + STZ (60 mg/kg) + cynaroside (25 mg/kg)-treated group.

To further clarify whether cynaroside attenuates hepatocyte senescence in NASH-T2DM by modulating HMGCS2-regulated ketone body production, we used adeno-associated virus (AAV8)-shHMGCS2 to treat the mouse model of NASH + T2DM (Figure 7A). In vivo bioluminescence imaging confirmed the predominant hepatic localization of HMGCS2 (Figure 7B), and Western blot analysis confirmed the effective silencing of HMGCS2-targeted constructs, specifically in liver tissue, in contrast to inefficient interference observed in cardiac and renal tissues (Figure 7C and Figure S3). Serum analyses revealed significantly upregulated AcAc and BHB levels in model mice compared to cynaroside-treated animals (Figure 7D). Notably, HMGCS2 inhibition reduced ketone body concentrations, indicating that HMGCS2 knockdown attenuated cynaroside’s therapeutic effect on ketone body overproduction in NASH-T2DM C57BL/6J mice.

SA-β-gal staining (Figure 7E) and the quantification of senescence markers p53 and p21 (Figure 7F,G) demonstrated significantly reduced hepatocyte senescence in cynaroside-treated mice relative to model controls. Conversely, HMGCS2 shRNA intervention increased senescence markers compared to cynaroside monotherapy. Notably, HMGCS2 knockdown weakened the inhibition effect of cynaroside on intracellular ROS levels in CDAHFD + STZ-treated mice (Figure S4). These findings collectively revealed that HMGCS2 knockdown compromised cynaroside’s effects on hepatocyte senescence in NASH-T2DM mice. Analysis of serum biomarkers showed significant alterations in liver injury and lipid metabolism parameters (Figure 7H,I). Relative to the model group, cynaroside (50 mg/kg) administration substantially attenuated serum levels of hepatic injury markers (ALT, AST) and dyslipidemia indicators (TC, TG). Conversely, concomitant HMGCS2 shRNA intervention abrogated these ameliorative effects, yielding significantly elevated biomarker levels compared to cynaroside monotherapy. Histopathological evaluation via H&E staining demonstrated a marked reduction in hepatic lipid accumulation and inflammatory infiltration in cynaroside-treated mice. However, HMGCS2 knockdown in cynaroside-administered mice significantly exacerbated these pathological features relative to the cynaroside-only group (Figure 7J). Taken together, knockdown of HMGCS2 attenuated cynaroside’s effects on liver injury and dyslipidemia in C57BL/6J mice with NASH combined with T2DM.

## 4. Discussion

NAFLD encompasses a spectrum of hepatic pathologies ranging from simple steatosis to NASH and fibrosis, culminating in elevated risks of cirrhosis and hepatocellular carcinoma [19,20]. Crucially, NAFLD exhibits a strong clinical association with insulin resistance and T2DM [21,22,23]. To investigate the potential promotive effect of T2DM on NAFLD progression, we established two experimental murine models: (1) a standalone NAFLD model induced by a 45% high-fat methionine–choline-deficient diet supplemented with 1% cholesterol (CDAHFD) and (2) a NAFLD-T2DM comorbidity model generated through triple intraperitoneal injections of streptozotocin (STZ; 60 mg/kg) combined with CDAHFD feeding. It has been reported that a high-fat diet and low-dose STZ (30 mg/kg/d for 3 days) injections in male mice can cause fasting hyperglycemia, fasting hyperinsulinemia, and insulin resistance, which can be used to establish a model of type 2 diabetes [24]. To determine the optimal STZ concentration for establishing the type II diabetes model, we conducted a dose–response study based on a literature review. STZ was administered at doses of 30, 45, 60, and 75 mg/kg per day via three consecutive injections. Fasting blood glucose levels were monitored to assess the induction of T2DM. The dose of 60 mg/kg per day for three days was ultimately selected, as it consistently produced stable hyperglycemia. Blood glucose analyses revealed that after 6 weeks of CDAHFD intervention, fasting blood glucose levels were significantly higher than 6.1 mmol/L. Notably, mice receiving concurrent STZ and CDAHFD exhibited hyperglycemia exceeding 11.1 mmol/L within 6 weeks of dietary induction, with levels significantly surpassing those in CDAHFD-only cohorts, demonstrating its reliability for establishing a robust type II diabetic mouse model. A longitudinal assessment demonstrated progressively elevated serum biomarkers of hepatic injury and dyslipidemia in NAFLD mice over time. Concomitant T2DM exacerbated these pathological manifestations, as evidenced by significantly amplified liver damage and lipid metabolic derangements. Histopathological analysis further revealed the substantial accumulation of senescent cells within hepatic tissues, with markedly intensified senescence observed in NAFLD-T2DM comorbid models relative to NAFLD counterparts. During NAFLD progression from steatosis to steatohepatitis, the hepatic expression of SASP factors exhibits progressive up-regulation. Notably, this SASP induction is significantly amplified in NAFLD-T2DM comorbidity models. Key SASP components—specifically IL-1β, IL-6, IL-16, ICAM-1, and ICAM-2—demonstrated a mechanistic role in exacerbating hepatic inflammation, thereby accelerating the onset and progression of pro-inflammatory cascades in experimental models of NAFLD.

To elucidate the mechanistic basis for exacerbated hepatic injury and accelerated cellular senescence in NAFLD-T2DM comorbid models relative to NAFLD alone, we analyzed ketone body production. Analyses revealed a significant elevation in ketone bodies in NAFLD mice during weeks 0–4, with markedly amplified levels in NAFLD-T2DM cohorts. Paradoxically, by week 10, NAFLD-T2DM mice exhibited a pronounced decline in ketone bodies, which fell significantly below those of NAFLD-only controls. The subsequent investigation of HMGCS2—the rate-limiting enzyme in ketogenesis—demonstrated congruent temporal dynamics: (1) during weeks 0–4, hepatic HMGCS2 expression was substantially upregulated in NAFLD-T2DM mice compared to the NAFLD controls, exhibiting progressive elevation over time, and (2) beyond week 6, HMGCS2 expression underwent significant suppression in the NAFLD-T2DM models compared to the NAFLD counterparts and continued to decline until the 10th week. Collectively, these findings suggest that the dysregulation of ketogenesis may play an important role in the accelerated progression of liver disease in NAFLD combined with T2DM.

To investigate the mechanistic role of HMGCS2 in ketogenesis dysregulation associated with NASH-T2DM comorbidity, we performed HMGCS2 overexpression via the tail vein injection of AAV8 vectors encoding HMGCS2 in C57BL/6J mice. Key findings demonstrated that (1) HMGCS2 overexpression effectively normalized aberrant ketone body production in NASH-T2DM mice; (2) this intervention significantly attenuated hepatocyte senescence, as evidenced by reduced SA-β-gal-positive cells and downregulated senescence-associated proteins; and (3) a concomitant amelioration of histopathological injury and inflammatory infiltration was observed.

Despite the current paucity of effective pharmacological agents for NAFLD in clinical practice, traditional Chinese medicine has demonstrated significant therapeutic potential [25,26]. In this study, we investigated the therapeutic effects of cynaroside, a natural flavonoid, in the model of NASH + T2DM induced by CDAHFD and STZ. Drawing upon the established literature [27] and prior experimental evidence, we administered three distinct dosage regimens of cymaroside (12.5, 25, and 50 mg/kg) in murine models to assess the pharmacological efficacy. Cynaroside administration significantly attenuated hepatic injury and suppressed inflammatory infiltration in mice with NAFLD-T2DM comorbidity. Pathogenetically, NASH constitutes an inflammatory hepatic disorder driven principally by cellular lipotoxicity. Hepatocytes under lipotoxicity exhibit cardinal features of metabolic stress—including mitochondrial dysfunction, DNA damage, and telomere attrition—which activate the cellular senescence program [28,29]. During NASH progression, senescent hepatocytes accumulated in parallel with inflammatory exacerbation, establishing a causal linkage between persistent hepatocyte senescence and maladaptive tissue repair responses that ultimately precipitate fulminant hepatic failure [30,31]. To elucidate the mechanistic contribution of cellular senescence to cynaroside’s therapeutic efficacy in NASH-T2DM comorbidity, we quantitatively assessed hepatic senescence markers via SA-β-gal staining and senescence regulators p53 and p21. Notably, cynaroside treatment induced the dose-dependent attenuation of hepatocyte senescence, as evidenced by a reduction in SA-β-gal-positive cells and the downregulation of p53 and p21 expression. Substantial evidence links cellular senescence to intracellular ROS accumulation [32,33]. Accordingly, we quantified hepatic ROS levels, demonstrating that cynaroside significantly suppressed ROS generation in hepatocytes compared to the CDAHFD + STZ model group.

Ketone bodies principally comprise AcAc, BHB, and acetone. Ketogenesis disposes of up to two-thirds of the lipids that enter the liver, and its dysregulation significantly contributes to NAFLD pathogenesis [12,34]. To investigate the mechanistic link between ketogenesis modulation and cynaroside’s anti-senescence effects in NASH-T2DM comorbidity, serum concentrations of AcAc and BHB were quantified. Analytical data revealed that circulating AcAc and BHB levels were significantly decreased in NASH-T2DM mice compared to the controls. However, the restoration of ketone body levels was found in the cymaroside-treated group. A concomitant assessment of hepatic HMGCS2 expression demonstrated concordant up-regulation induced by cynaroside treatment. Collectively, these findings indicate that cynaroside attenuates hepatocyte senescence through the enhancement of HMGCS2 expression and subsequent ketogenesis, thereby ameliorating the pathological manifestations of NASH-T2DM. However, the precise mechanism through which HMGCS2-mediated ketogenesis influences ROS and thereby mediates cellular senescence requires further investigation.

To delineate the essential role of HMGCS2 in mediating cynaroside’s dual regulation of ketogenesis and cellular senescence suppression, we established an in vivo knockdown model via the tail vein injection of AAV8-delivered HMGCS2 shRNA (AAV8-shHMGCS2) in NASH-T2DM mice. Critical findings demonstrated that HMGCS2 knockdown (1) abrogated the cynaroside-induced normalization of serum ketone bodies; (2) attenuated cynaroside’s hepatoprotective effects, as evidenced by the reversal of ALT/AST reduction; (3) abolished the lipid-lowering efficacy; and (4) neutralized cynaroside’s anti-senescence activity, which manifested as follows: up-regulated the expression of p53 and p21, increased SA-β-gal-positive hepatocytes, and enhanced ROS production. In conclusion, the present study demonstrated that cynaroside promoted ketone body production in the liver through the up-regulation of HMGCS2, which in turn inhibited hepatocyte senescence in order to ameliorate NASH-combined T2DM and slow down the progression of liver disease.

## 5. Conclusions

In summary, the HMGCS2-mediated regulation of ketogenesis represented an important mechanism underlying the attenuation of liver disease progression in NASH-T2DM comorbidity. Our study provided an experimental basis on which cynaroside could be developed into a promising drug for the treatment of NASH combined with T2DM in the future.

## Data Availability

The data that support the findings of this study are available from the corresponding author upon reasonable request.

## Supplementary Materials

The following supporting information can be downloaded at: https://www.mdpi.com/article/10.3390/biom15081181/s1, Figure S1: Western blot analysis confirming HMGCS2 plasmid transfection efficiency in the heart and kidney; Figure S2: Dihydroethidium (DHE) probe was used to determine ROS production; Figure S3: The transfection efficiency of HMGCS2 shRNA in the heart and kidney was evaluated by western blot analysis; Figure S4: DHE probe was used to determine ROS production.

## Abbreviations

The following abbreviations are used in this manuscript:

AcAc	Acetoacetate
BHB	β-hydroxybutyrate
CDAHFD	A methionine–choline-deficient, high-fat diet supplemented with 1% cholesterol
EV	Empty vector
FBG	Fasting blood glucose
γ-H2AX	Phosphorylation of histone H2AX and gamma-H2AX
HCC	Hepatocellular carcinoma
H&E	Hematoxylin and eosin
HF	Hepatic fibrosis
HMGCS2	Hydroxymethylglutaryl-CoA synthase 2
HMG-CoA	Hydroxymethylglutaryl-CoA
IL-1β	Interleukin-1 beta
NAFLD	Non-alcoholic fatty liver disease
NASH	Non-alcoholic steatohepatitis
PA	Palmitic acid
ROS	Reactive oxygen species
SA-β-gal	Senescence-associated β-galactosidase
SASP	Senescence-associated secretory phenotype
shRNA	Short hairpin RNA
STZ	Streptozotocin
T2DM	Type 2 diabetes mellitus
TNF-α	Tumor necrosis factor-α
