# Supplementary material for: Targeting HMGCS2: Ketogenesis Suppression Accelerates NAFLD Progression in T2DM Comorbidity, While Cynaroside Ameliorates NASH in Concomitant T2DM
Source: Biomolecules. 2025 Aug 18;15(8):1181. doi: 10.3390/biom15081181 (PMC12385132; doi:10.3390/biom15081181)
Supplement: Supplementary file 1 [file biomolecules-15-01181-s001.zip › Supporting Information for Original article.pdf]

Supporting Information for Original article

Targeting HMGCS2: Ketogenesis Suppression Accelerates NAFLD Progression in T2DM Comorbidity, While Cynaroside Ameliorates NASH in Concomitant T2DM

Yongsheng Shu [1], Wanqing Shen [1], Wanyu Feng [1], Meijun Pan [1], Xinyi Xu [1], Shuguo Zheng [1,2,*] and Huanhuan Jin [1,2,*]

[1] Department of Pharmacology, School of Pharmacy, Wannan Medical College, Wuhu 241002, China; 20249138@stu.wnmc.edu.cn (Y.S.); 20229074@stu.wnmc.edu.cn (W.S.); 20239135@stu.wnmc.edu.cn (W.F.); 20239130@stu.wnmc.edu.cn (M.P.); 22107080042@stu.wnmc.edu.cn (X.X.)

[2] Laboratory of Pharmacology of Chinese Medicine, School of Pharmacy, Wannan Medical College, Wuhu 241002, China

* Correspondence: zhengsg2000@wnmc.edu.cn (S.Z.); huanhuanjin@wnmc.edu.cn (H.J.)

Supporting figures

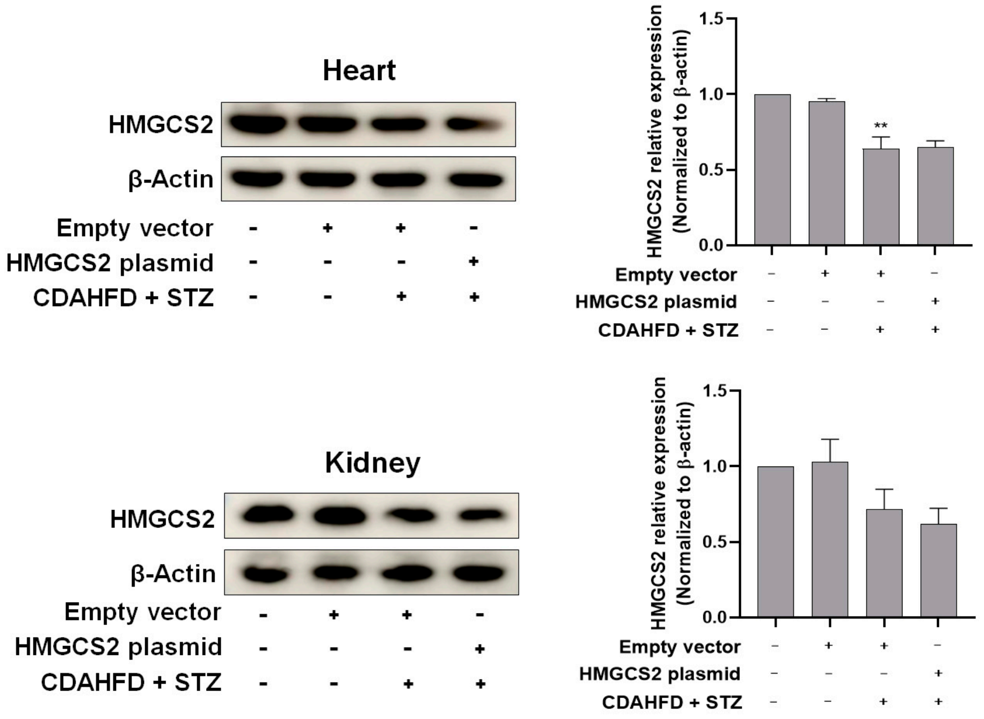

Figure S1 Western blot analysis confirming HMGCS2 plasmid transfection efficiency in the heart and kidney ($n = 3$). Significance: $^{**}p < 0.01$ vs. Empty vector (EV) group.

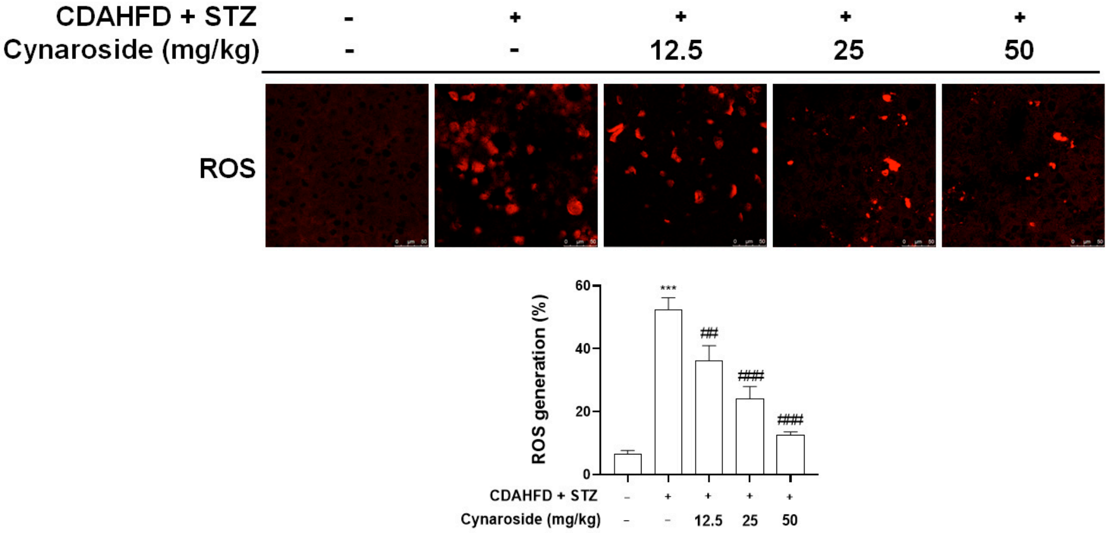

Figure S2 Dihydroethidium (DHE) probe was used to determine ROS production (n = 3). Representative images of ROS staining of liver sections are shown. Scale bars, 50 μm. Bar graphs showed the percentage quantitation of ROS generation area using the software Image J as shown. Significance: $^{***}p < 0.001$ vs. NC group; $^{\#\#}p < 0.01$, $^{\#\#\#}p < 0.001$ vs. CDAHFD + STZ (60 mg/kg) group.

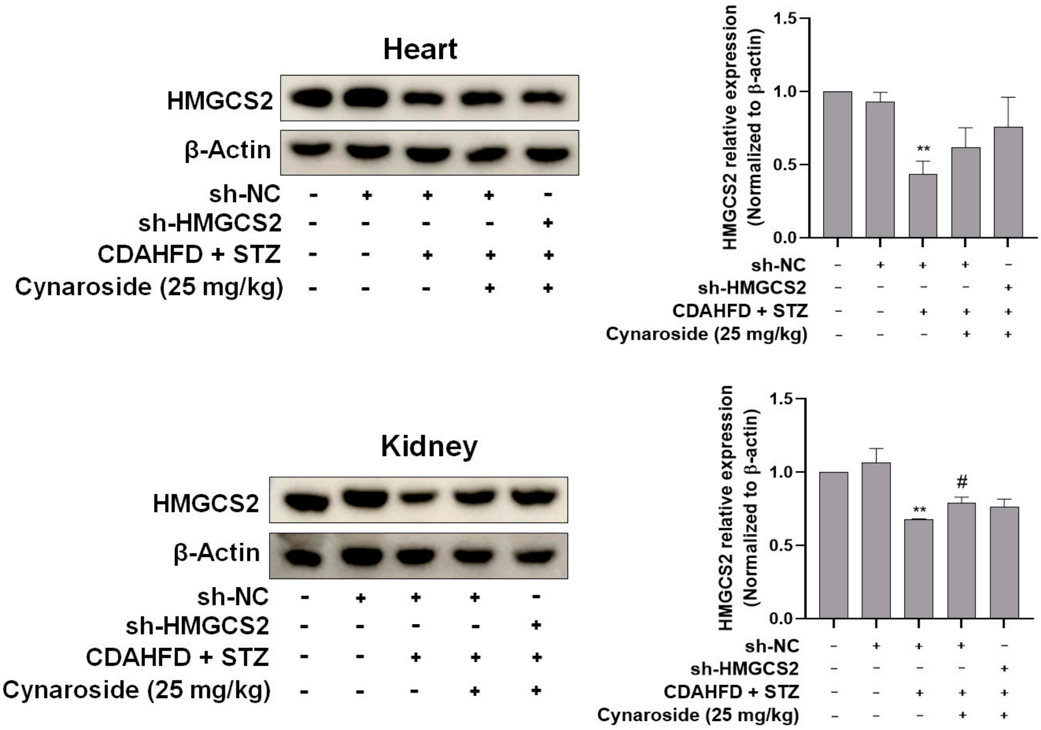

Figure S3 The transfection efficiency of HMGCS2 shRNA in the heart and kidney was evaluated by western blot analysis (n = 3). Significance: $^{**}p < 0.01$ vs. sh-NC group; $^{\#}p < 0.05$ vs. sh-NC + CDAHFD + STZ (60 mg/kg)-treated group.

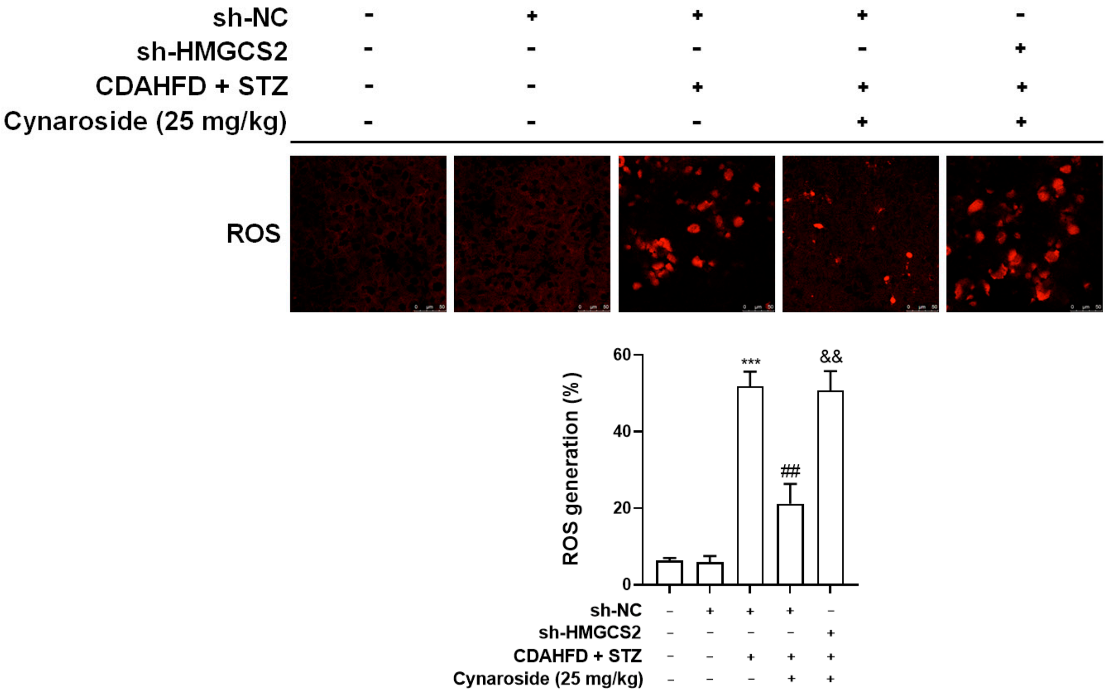

Figure S4 DHE probe was used to determine ROS production (*n* = 3). Representative images of ROS staining of liver sections are shown. Scale bars, 50 μm. Significance: $^{***}p < 0.001$ vs. sh-NC group; $^{\#\#}p < 0.01$ vs. sh-NC + CDAHFD + STZ (60 mg/kg)-treated group; $^{\&\&}p < 0.01$ vs. sh-NC + CDAHFD + STZ (60 mg/kg) + cynaroside (25 mg/kg)-treated group.
