# Supplementary figures and images for: Targeting HMGCS2: Ketogenesis Suppression Accelerates NAFLD Progression in T2DM Comorbidity, While Cynaroside Ameliorates NASH in Concomitant T2DM
Source: Biomolecules. 2025 Aug 18;15(8):1181. doi: 10.3390/biom15081181 (PMC12385132; doi:10.3390/biom15081181)

Figure 3C

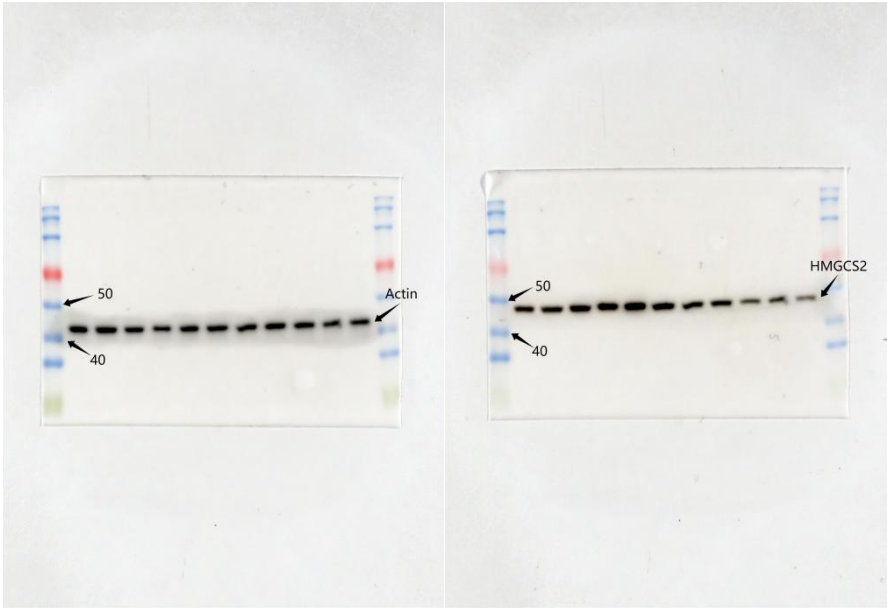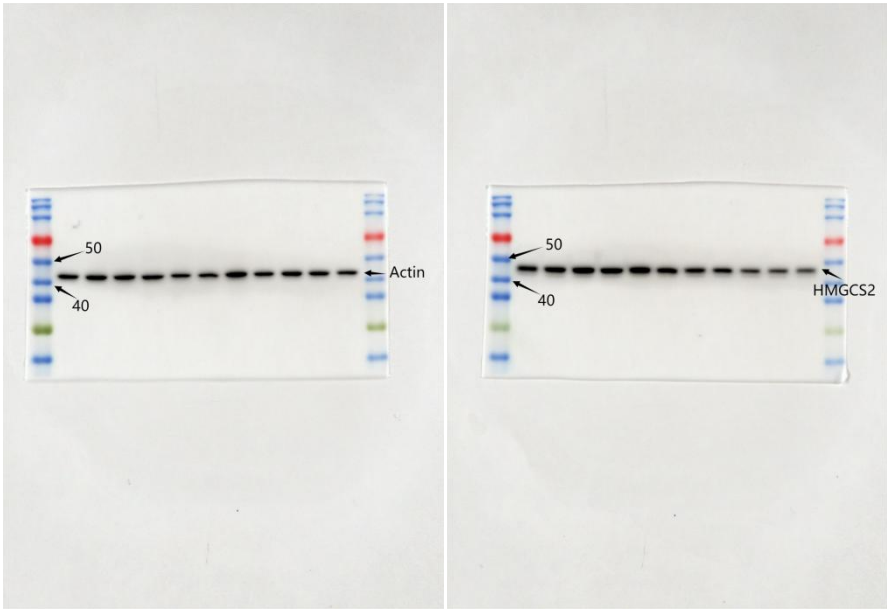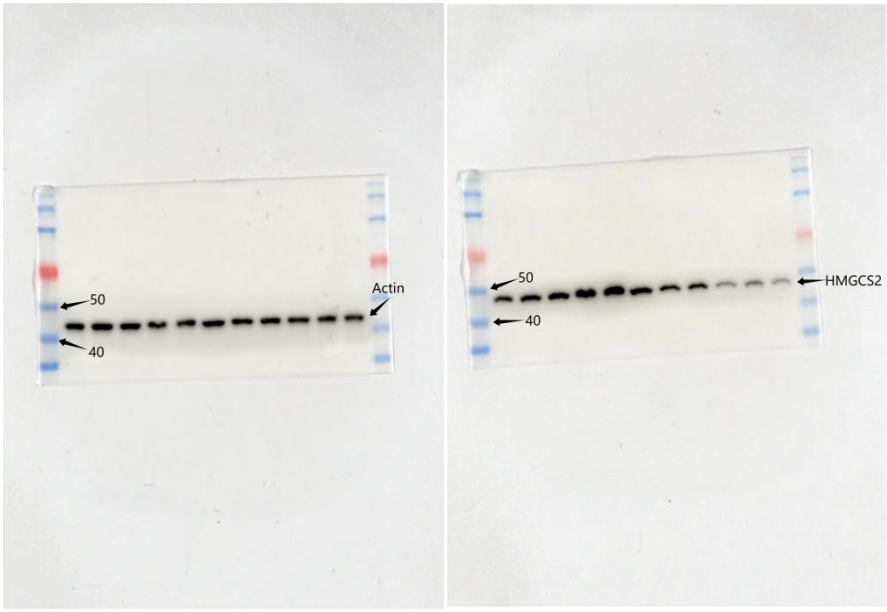

Figure 3D

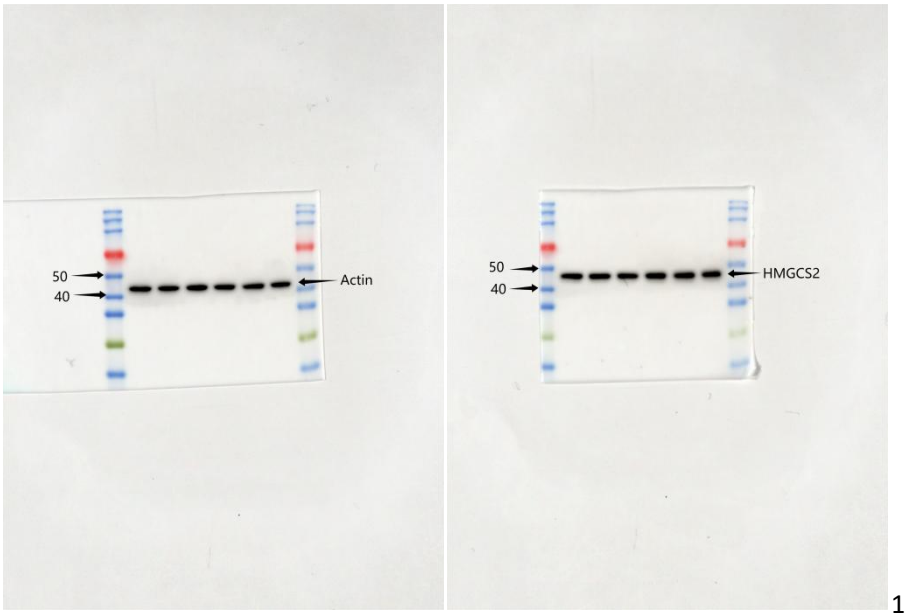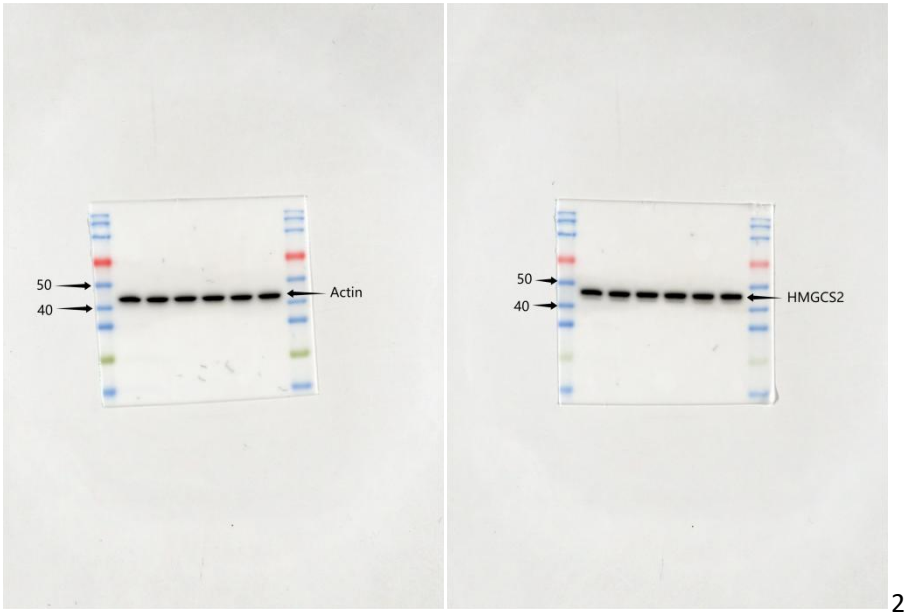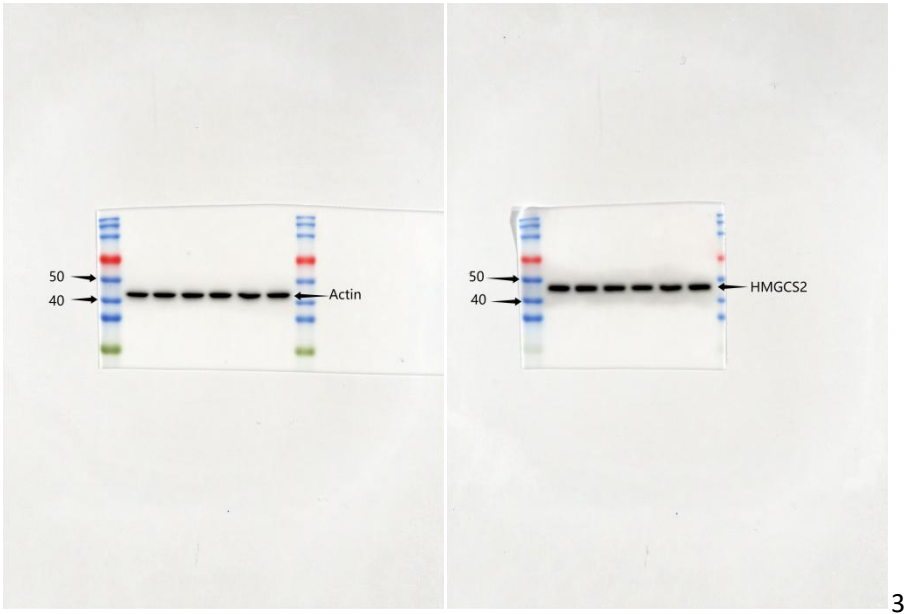

Figure 4B

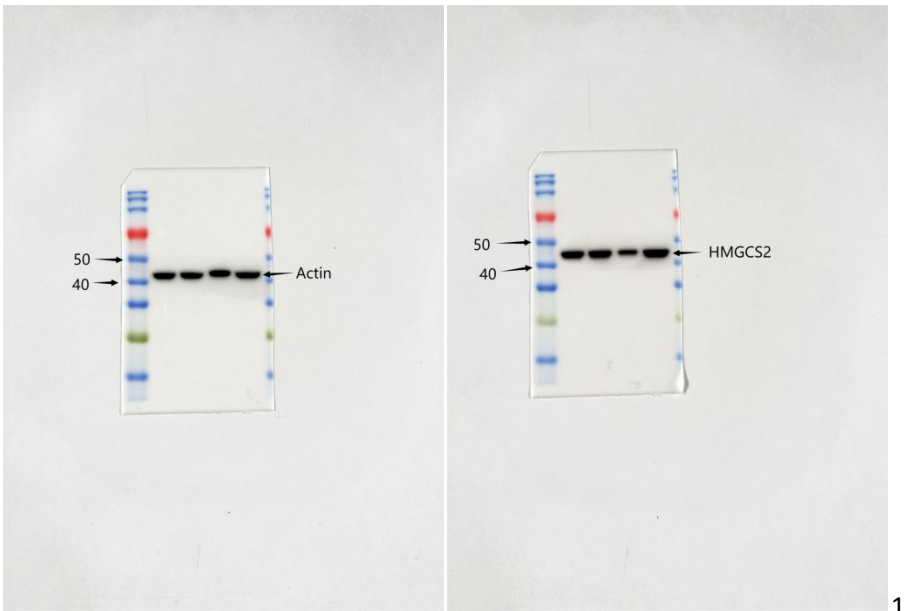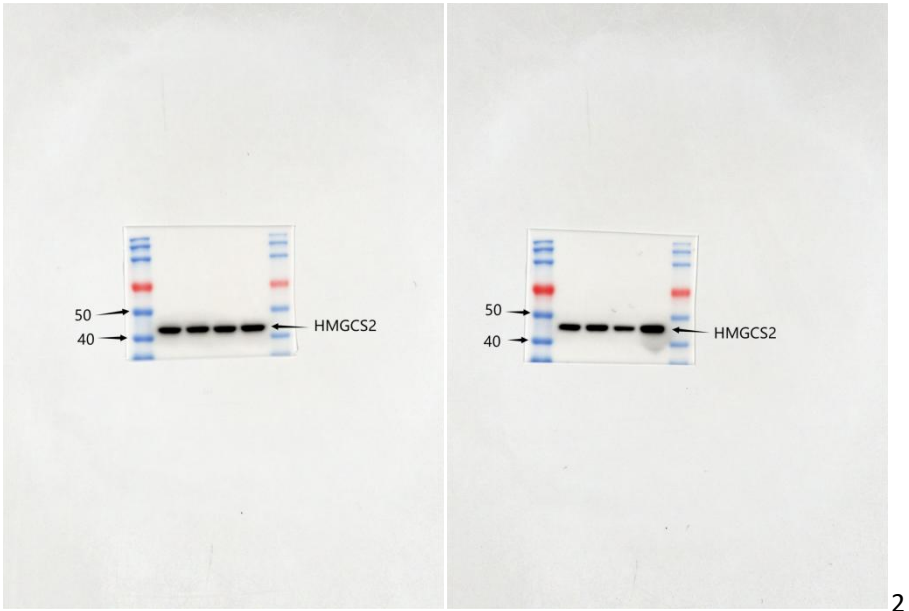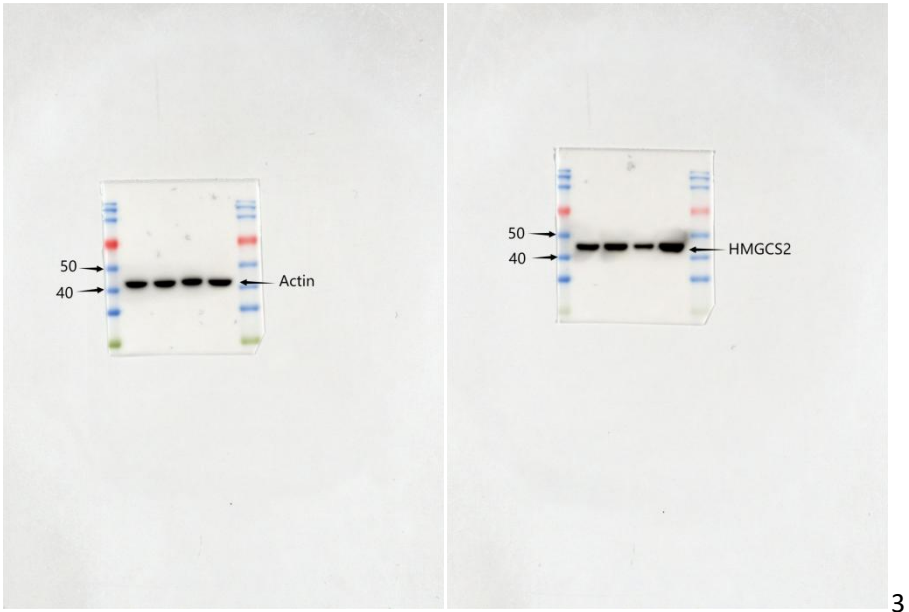

Figure 4G

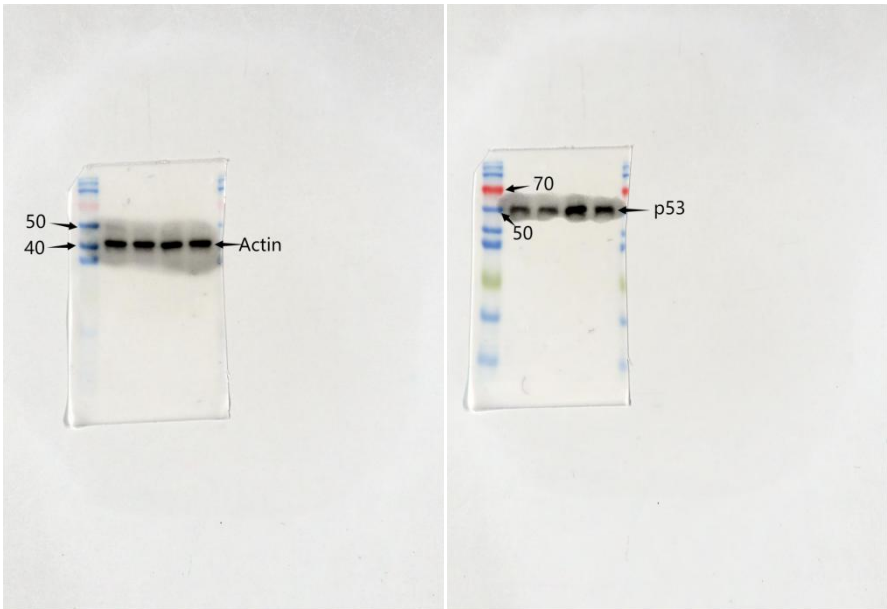

1

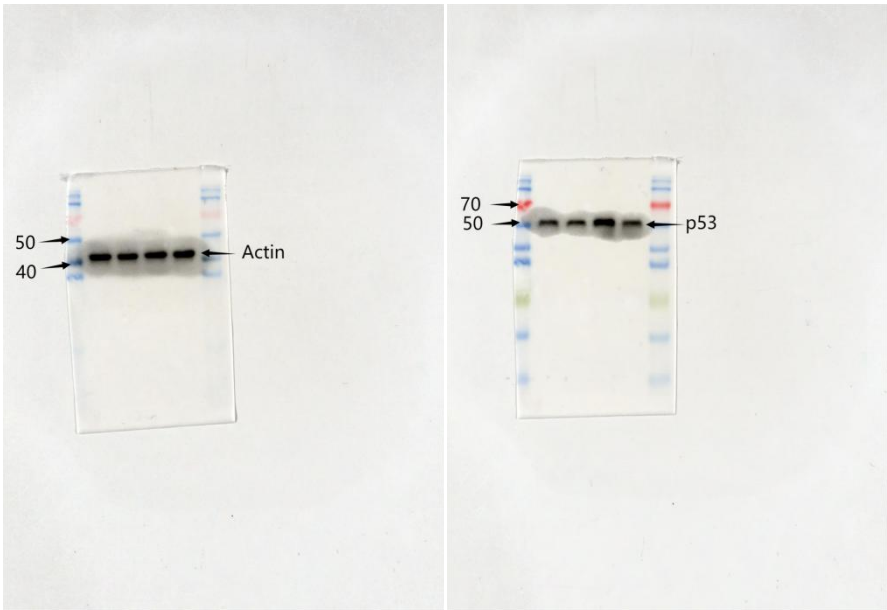

2

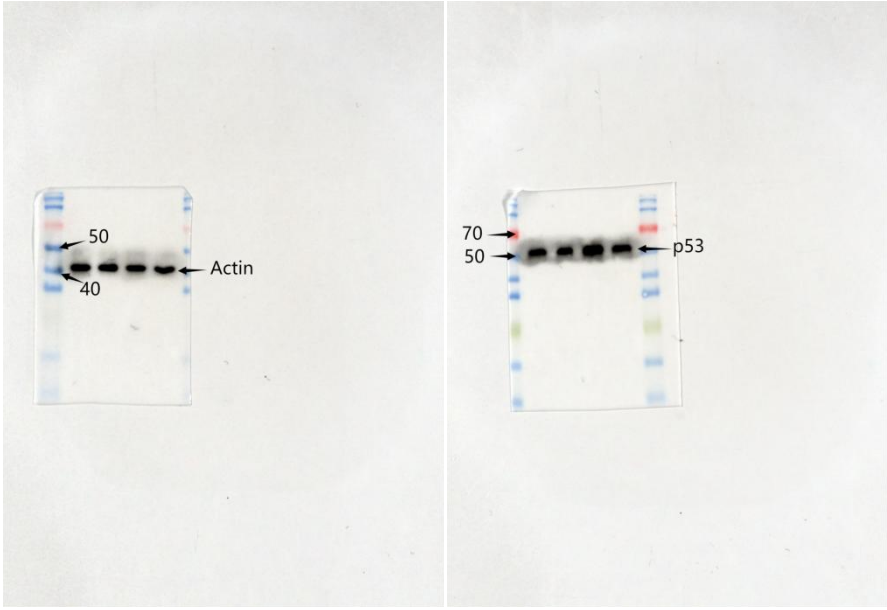

3

Figure 4H

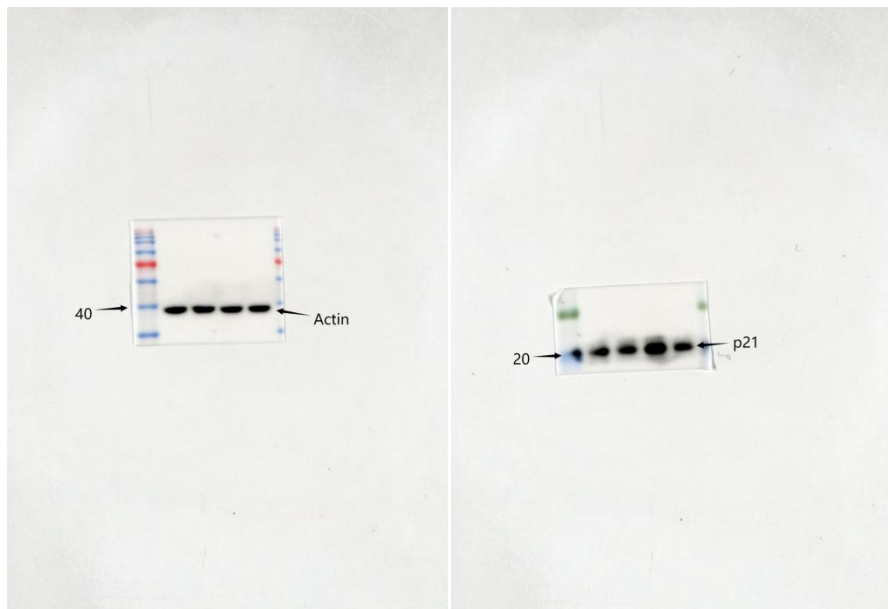

1

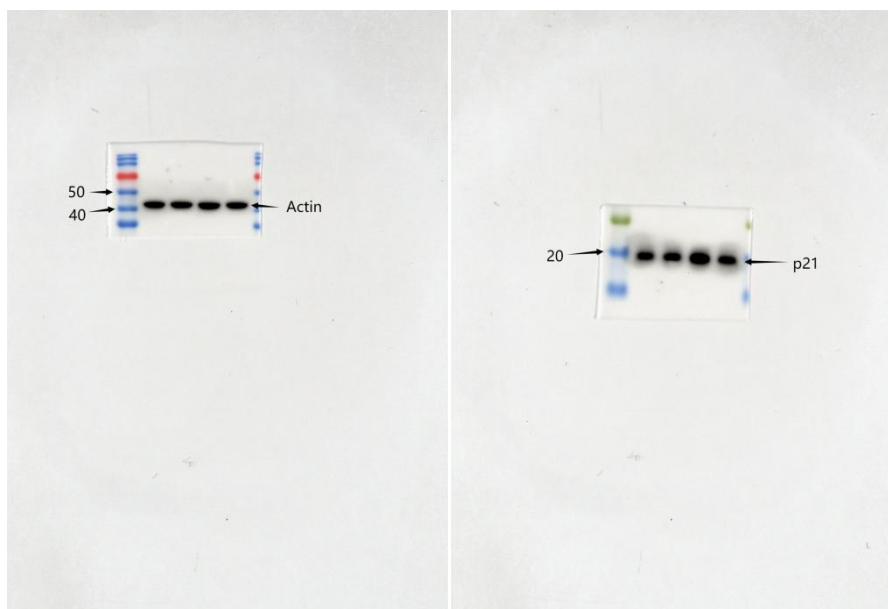

2

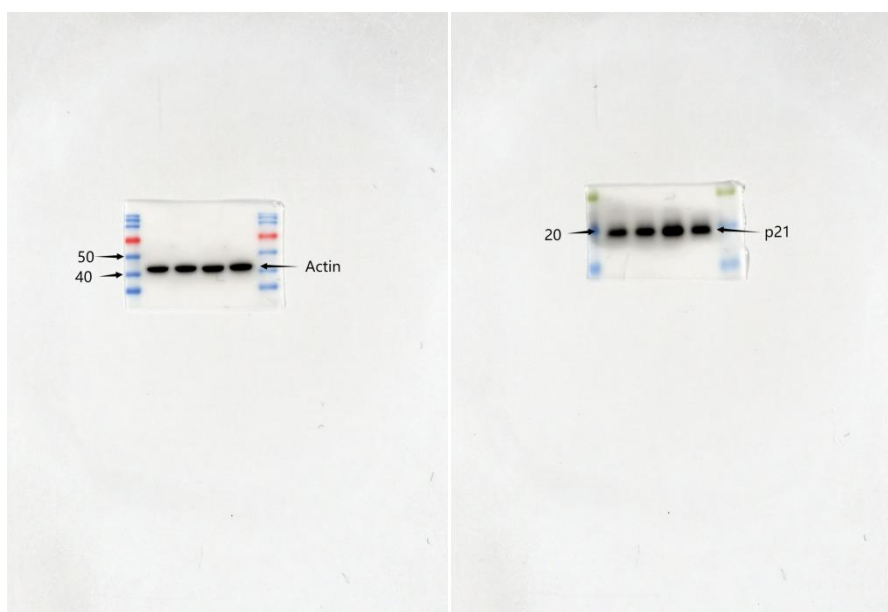

3

Figure 6B

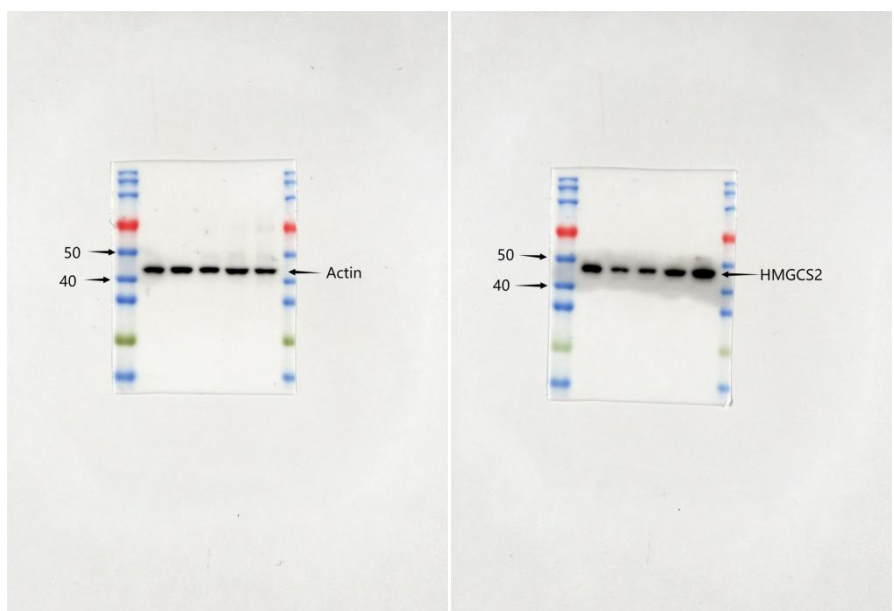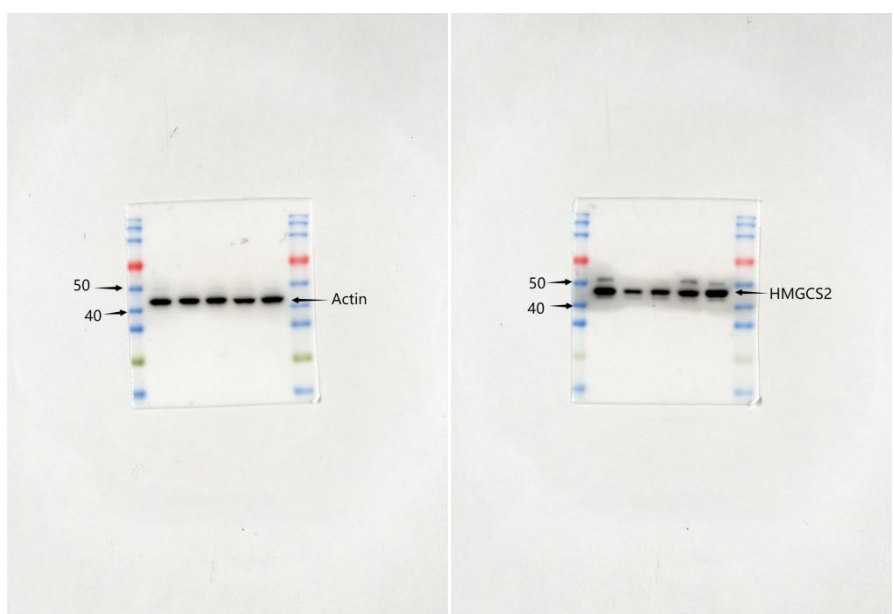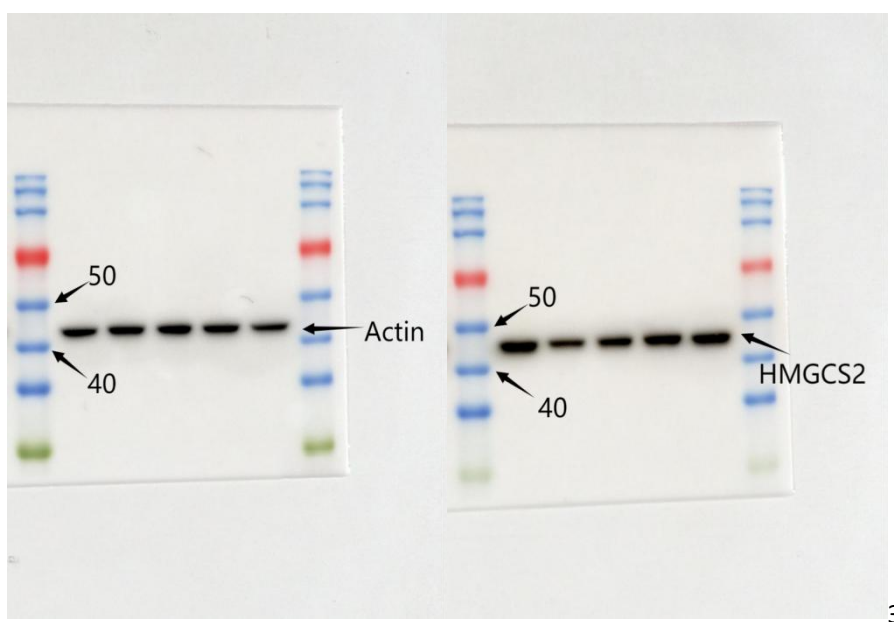

Figure 6E

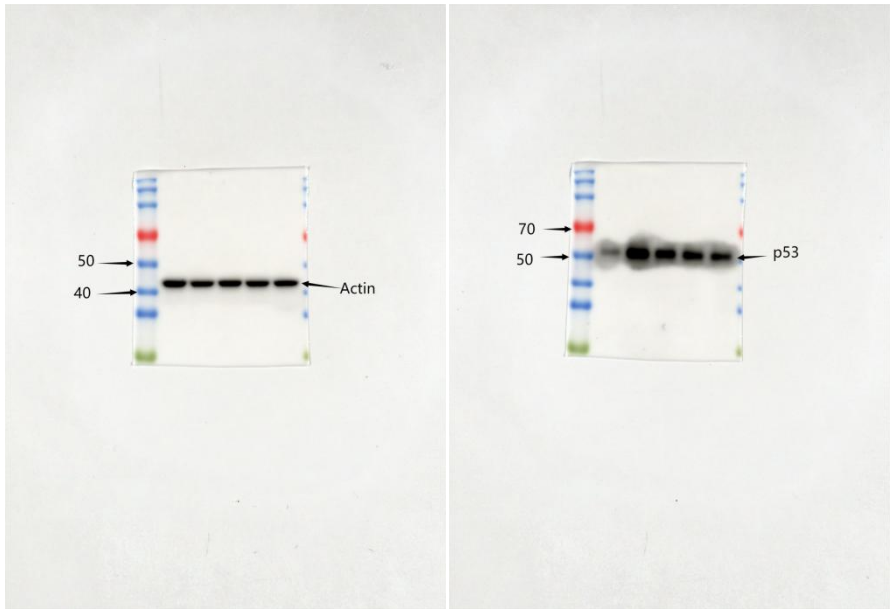

1

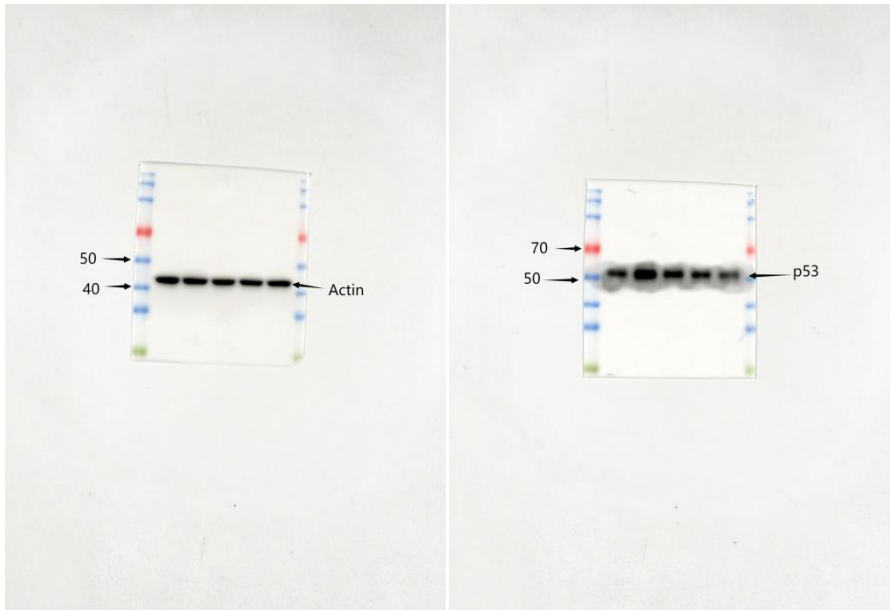

2

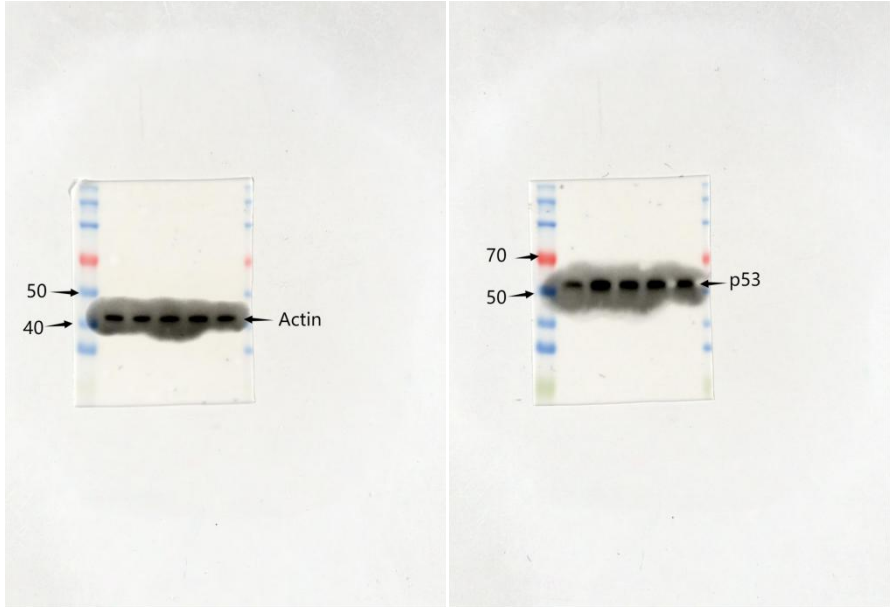

3

Figure 6F

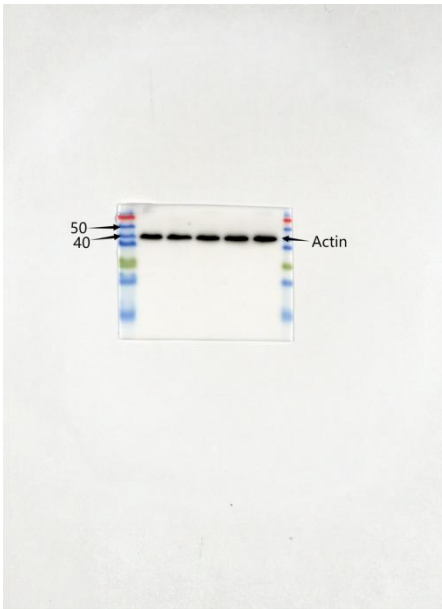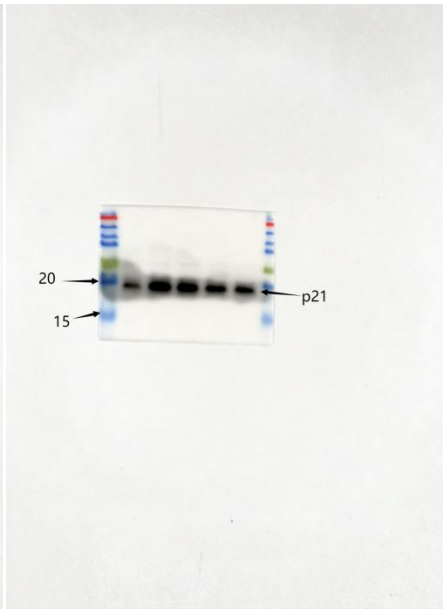

1

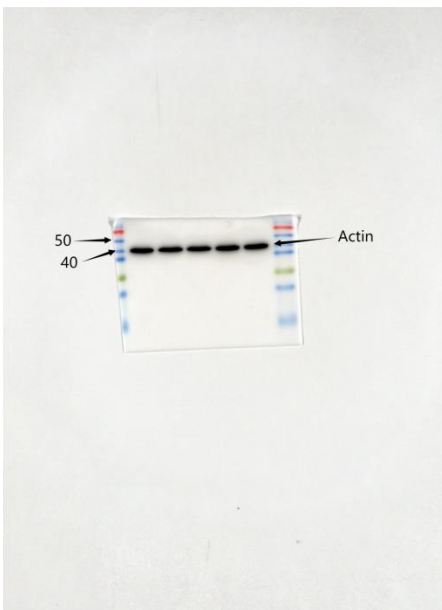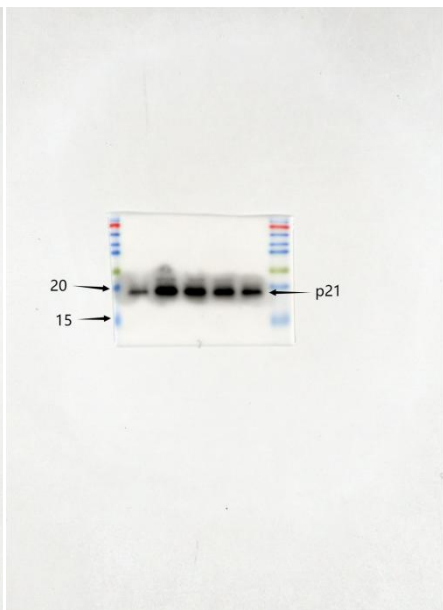

2

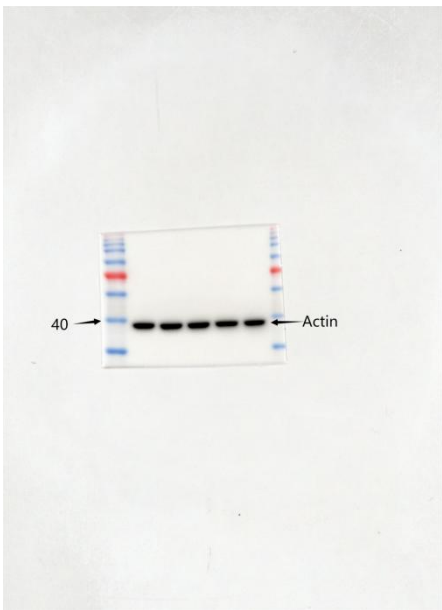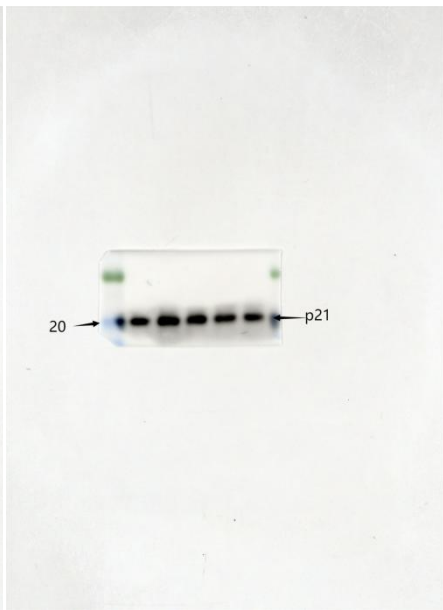

3

Figure 7B

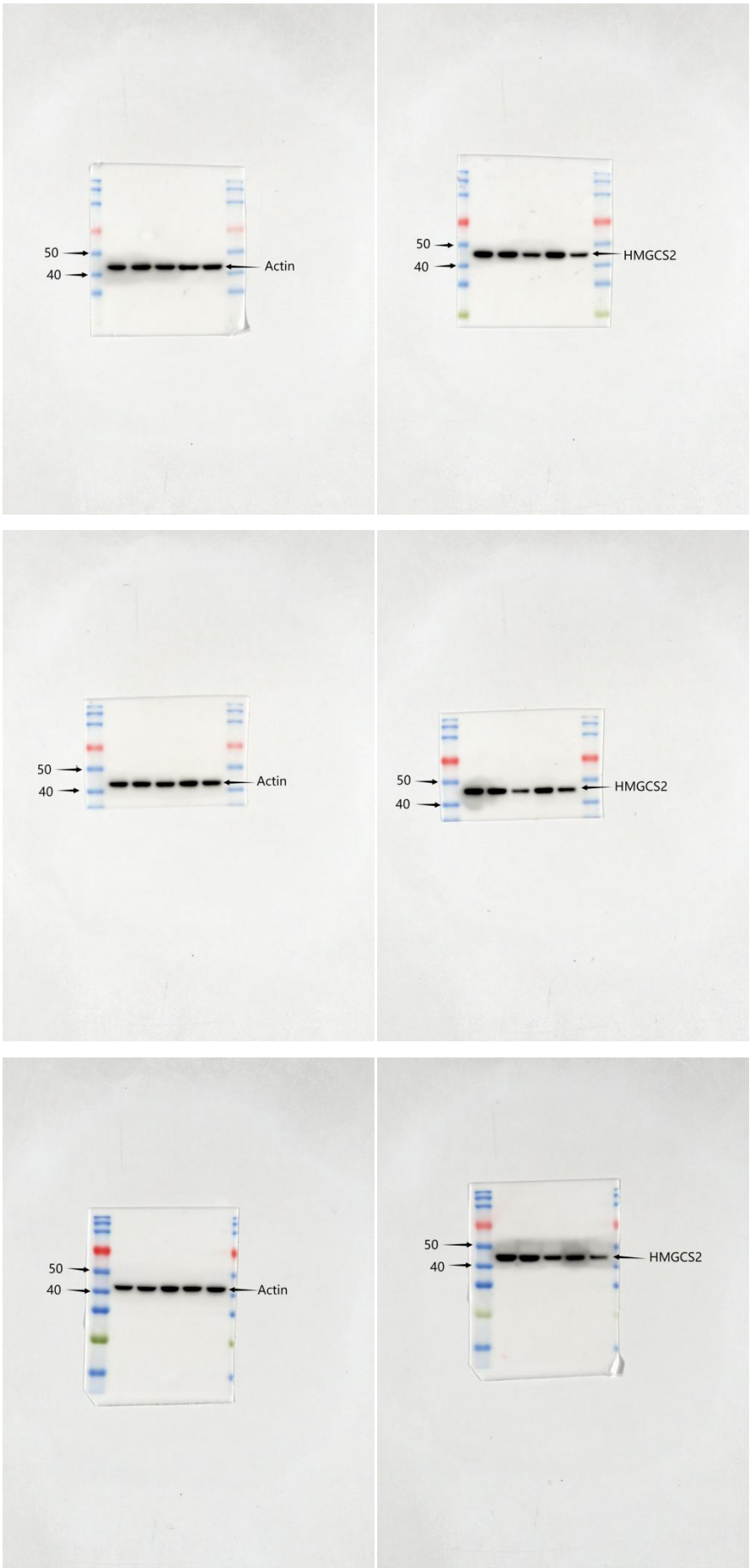

Figure 7E

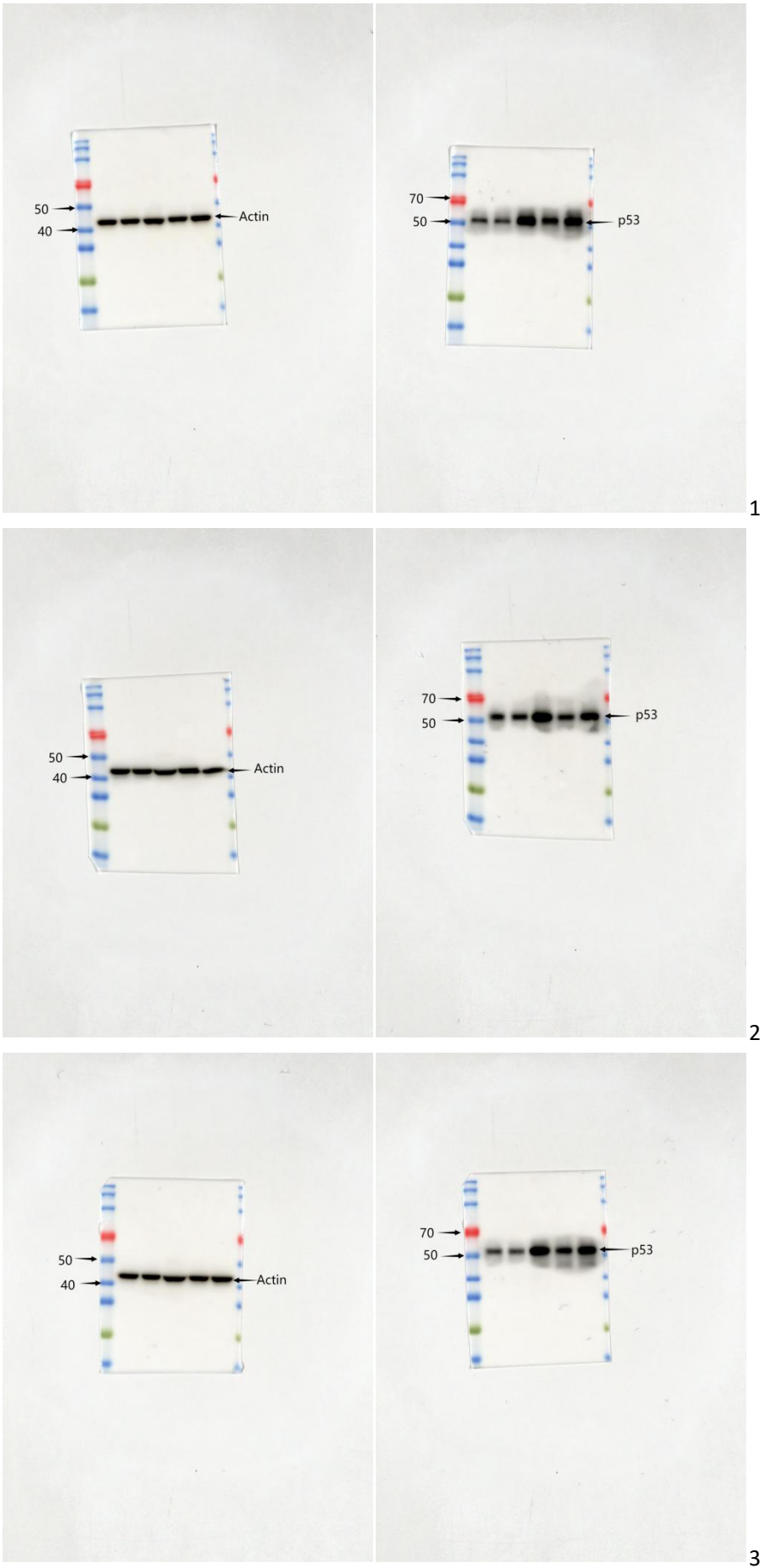

Figure 7F

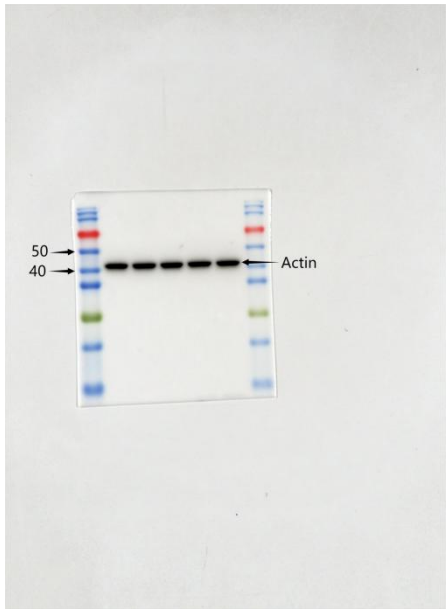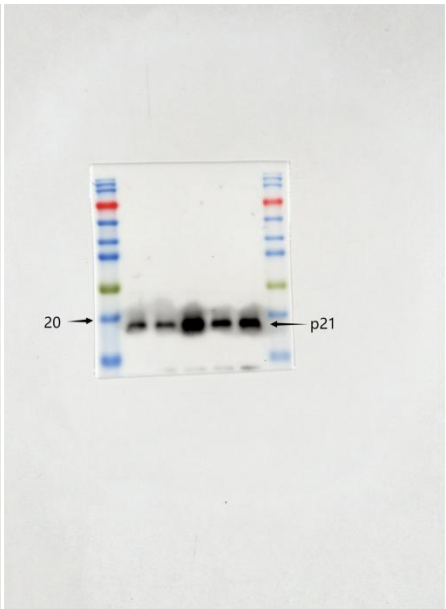

1

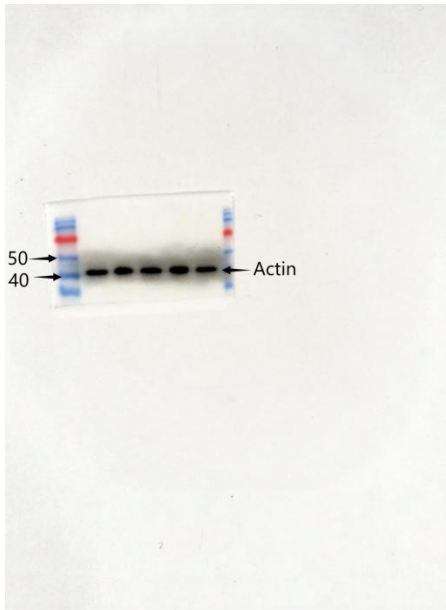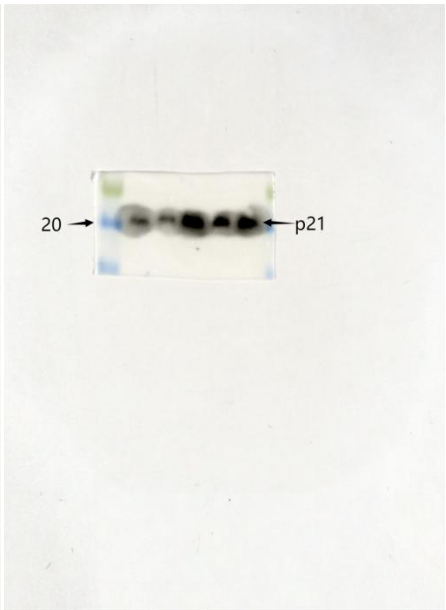

2

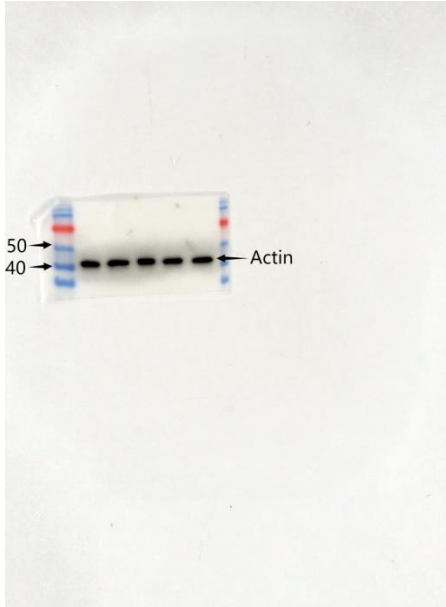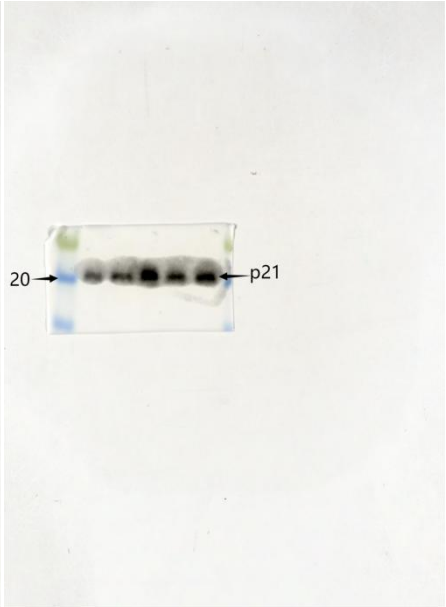

3

Supplement: Supplementary file 1 [file biomolecules-15-01181-s001.zip › biomolecules-3797587-image.pdf]
